# Flexible Mechanical Response Device With Optical Logic Emission Enabled by Synergistic Crystallization Engineering of Ester Polymer and Perovskite

**DOI:** 10.1002/advs.202508812

**Published:** 2025-08-13

**Authors:** Zhen‐Li Yan, Tzu‐Ming Hsu, Chien‐Hsin Wu, Jean‐Sebastien Benas, Ying‐Chi Huang, Wei‐Cheng Chen, Bi‐Hsuan Lin, Mei‐Hsin Chen, Ja‐Hon Lin, Hsinhan Tsai, Chu‐Chen Chueh, Chihaya Adachi, Ru‐Jong Jeng, Chi‐Ching Kuo

**Affiliations:** ^1^ Institute of Polymer Science and Engineering, Advanced Research Center for Green Materials Science and Technology National Taiwan University Taipei 10617 Taiwan; ^2^ Institute of Organic and Polymeric Materials National Taipei University of Technology Taipei 10608 Taiwan; ^3^ Advanced Research Center for Green Materials Science and Technology National Taiwan University Taipei 10617 Taiwan; ^4^ Department of Chemical Engineering National Taiwan University Taipei 10617 Taiwan; ^5^ National Synchrotron Radiation Research Center 101 Hsin‐Ann Road, Hsinchu Science Park Hsinchu 30076 Taiwan; ^6^ Department of Electro‐Optical Engineering National Taipei University of Technology Taipei 10608 Taiwan; ^7^ Department of Chemistry University of California Berkeley CA 94720 USA; ^8^ Department of Physics SUNY University at Buffalo Buffalo NY 14260 USA; ^9^ Center for Organic Photonics and Electronics Research (OPERA) and Department of Applied Chemistry Kyushu University 744 Motooka, Nishi Fukuoka 819‐0395 Japan

**Keywords:** crystallization‐regulated engineering, enhanced energy transfer, flexible light‐emitting diodes, mechanical logic response, perovskite–polymer composites

## Abstract

The flexible mechanical response device (FMRD) is developed using a stretchable perovskite light‐emitting diode. The FMRD achieves optical logic light‐emitting properties through a synergetic crystallization strategy involving a highly crystalline ester‐based polymer (hc‐ester) and perovskite. Research indicates that hc‐ester polymer influences the crystal growth of perovskite via ion‐dipole interaction, resulting in “crystallized space confinement.” Perovskite space confinement further optimizes perovskite's sub‐dimensional crystal phase ratio, enhancing exciton transmission efficiency and luminescence performance. Additionally, hc‐ester enhances the surface morphology of perovskite films and lowers the electron trap density, demonstrating significant potential for use in optoelectronic devices. Regarding its application, FMRD can generate optical logic signals through external force deformation, such as bending and stretching, making it useful for motion capture and mechanical stress sensing. In the bent state, FMRD shows an increase in luminance, a blue shift in emission, and improved external quantum efficiency, making it suitable for dynamic analog signal source output. At the same time, its reversible spectral changes and consistent variations in luminescence make it exceptional for mechanical stress sensing applications. This research presents an innovative, flexible optoelectronic device technology solution and paves the way for new applications of perovskite composite materials in optical logic devices and intelligent sensing.

## Graphical abstract

This study presents a flexible mechanical response device (FMRD) with optical logic properties, developed via synergistic crystallization engineering of ester‐based polymers and perovskite. Ion‐dipole interactions and crystallized space confinement tune perovskite crystal dimensions, enhancing exciton transfer and emission. The FMRD enables motion capture and stress sensing through deformation‐induced luminescence changes, advancing perovskite‐polymer optoelectronics.

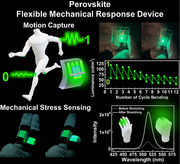

## 1 Introduction

The development of perovskite light‐emitting diodes (PeLEDs) has witnessed remarkable advancements in recent years,^[^ ^1^, ^2^, ^3^, ^4^ ^]^ driven by their exceptional optoelectronic properties such as high photoluminescence quantum yield (PLQY),^[^ ^5^, ^6^ ^]^ and tunable bandgaps.^[^ ^7^, ^8^, ^9^ ^]^ In particular, the emergence of flexible PeLEDs has opened new frontiers for next‐generation display and lighting applications.^[^ ^10^, ^11^, ^12^, ^13^, ^14^, ^15^, ^16^, ^17^, ^18^ ^]^ These devices, characterized by their mechanical flexibility, lightweight nature, and ability to conform to non‐planar surfaces, hold great promise for wearable electronics, foldable displays, and interactive lighting systems.^[^ ^19^, ^20^, ^21^ ^]^

One of the most promising applications of flexible LEDs is motion capture technology,^[^ ^22^, ^23^, ^24^ ^]^ where real‐time optical signal detection is crucial for fields such as human‐computer interaction,^[^ ^25^ ^]^ extended reality (XR),^[^ ^26^, ^27^ ^]^ and assistive communication tools.^[^ ^28^, ^29^, ^30^ ^]^ Traditional motion capture systems typically rely on external light sources and complex optical sensors, which limit their flexibility and applicability.^[^ ^31^, ^32^ ^]^ Integrating flexible PeLEDs with mechanical response capabilities introduces a novel approach to enhance motion capture accuracy through active dynamic optical signal generation.^[^ ^23^, ^24^, ^33^ ^]^ However, challenges such as generating identifiable light emissions during various mechanical deformations, ensuring consistent spectral output, and minimizing structural and electrical wearing degradation from repeated bending and stretching must be addressed for practical implementation.^[^ ^10^, ^21^ ^]^

Incorporating functional polymers capable of interacting with perovskite crystals has been explored to enhance the performance of perovskite optoelectronic devices.^[^ ^34^, ^35^, ^36^, ^37^, ^38^, ^39^, ^40^, ^41^, ^42^ ^]^ Polymers with strong ion‐dipole interactions can affect perovskite crystallization^[^ ^36^, ^39^ ^]^ defect passivation,^[^ ^34^, ^39^, ^41^, ^42^ ^]^ and overall film formation,^[^ ^35^, ^37^, ^40^ ^]^ improving optoelectronic properties.^[^ ^36^, ^37^, ^38^, ^41^ ^]^ While previous studies have investigated polymer‐perovskite interactions, the effect of polymer chain‐folding crystalline regions on these interactions and their subsequent influence on perovskite crystal growth remains largely unexplored. Understanding how polymer chain dynamics contribute to perovskite crystallization regulation is crucial for optimizing the optoelectronic performance of flexible PeLEDs.

In this study, we develop a synergistic crystallization strategy by inducing high‐crystallinity ester polymers (hc‐esters) into the perovskite system. This approach regulates the growth of perovskite crystals and enhances exciton energy transfer properties from sub‐dimensions to the main dimension structure. We demonstrate a flexible mechanical response device (FMRD) consisting of stretchable PeLEDs with optical logic emission by elucidating the relationship between polymer chain‐folding crystallinity, ion‐dipole interactions, and the dynamics of perovskite growth. This innovative design broadens the potential of flexible PeLEDs for motion capture and other interactive optoelectronic applications.

## 2 Results and Discussion

### 2.1 FMRD with Logical Emission Signals

**Figure 1:**
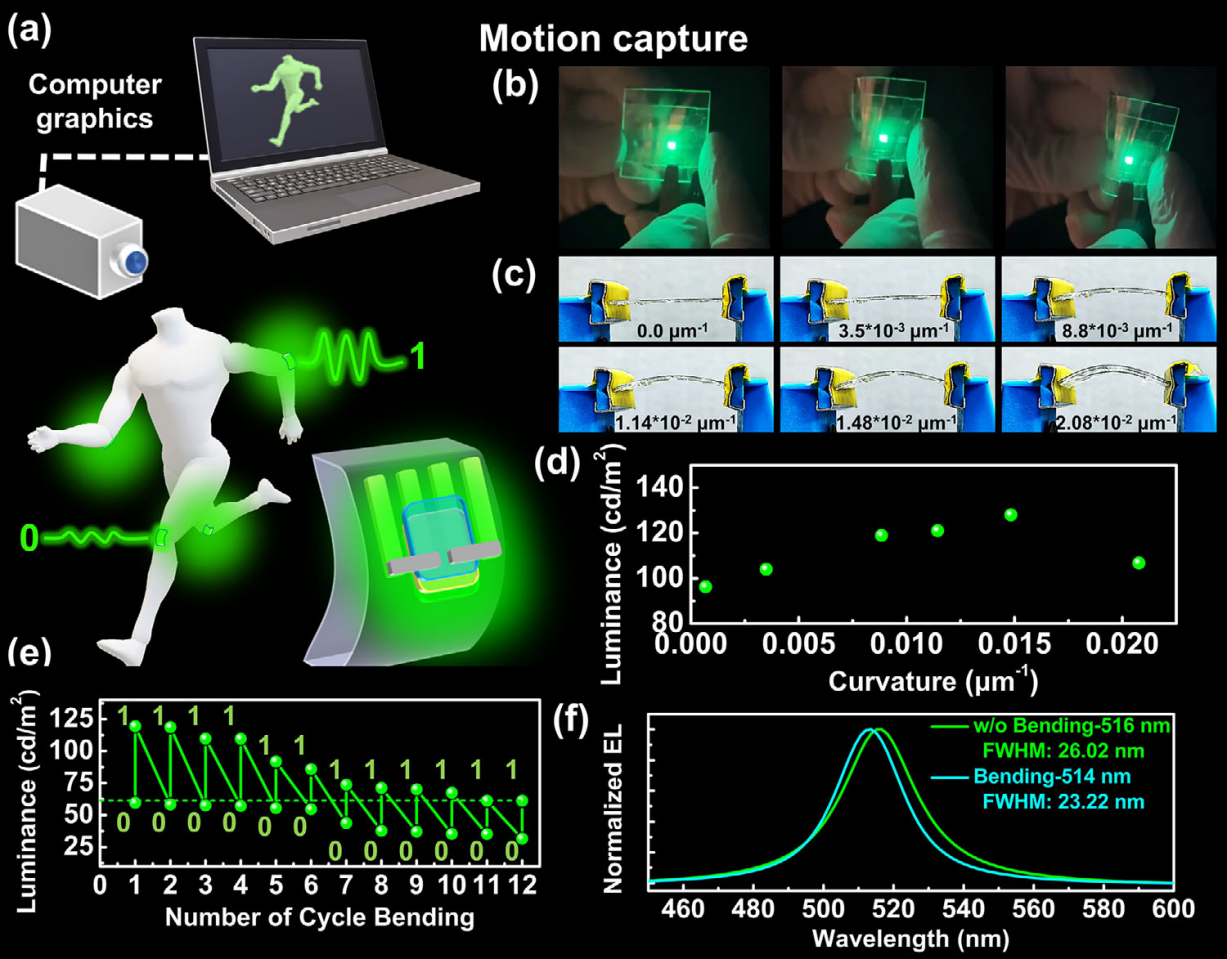
Application of ester‐based polymer‐perovskite composite in FMRD with logical emission signal for motion capture. a) Schematic diagram of motion capture application. b) The FMRD made of hc‐ester P.V.S.K. light up under bending. c) Under different bending curvatures, d) luminescence performance of FMRD at 4.5 V. e) Luminescence of FMRD under cyclic bending. f) EL spectrum of FMRD before and after bending.

**Figure 2:**
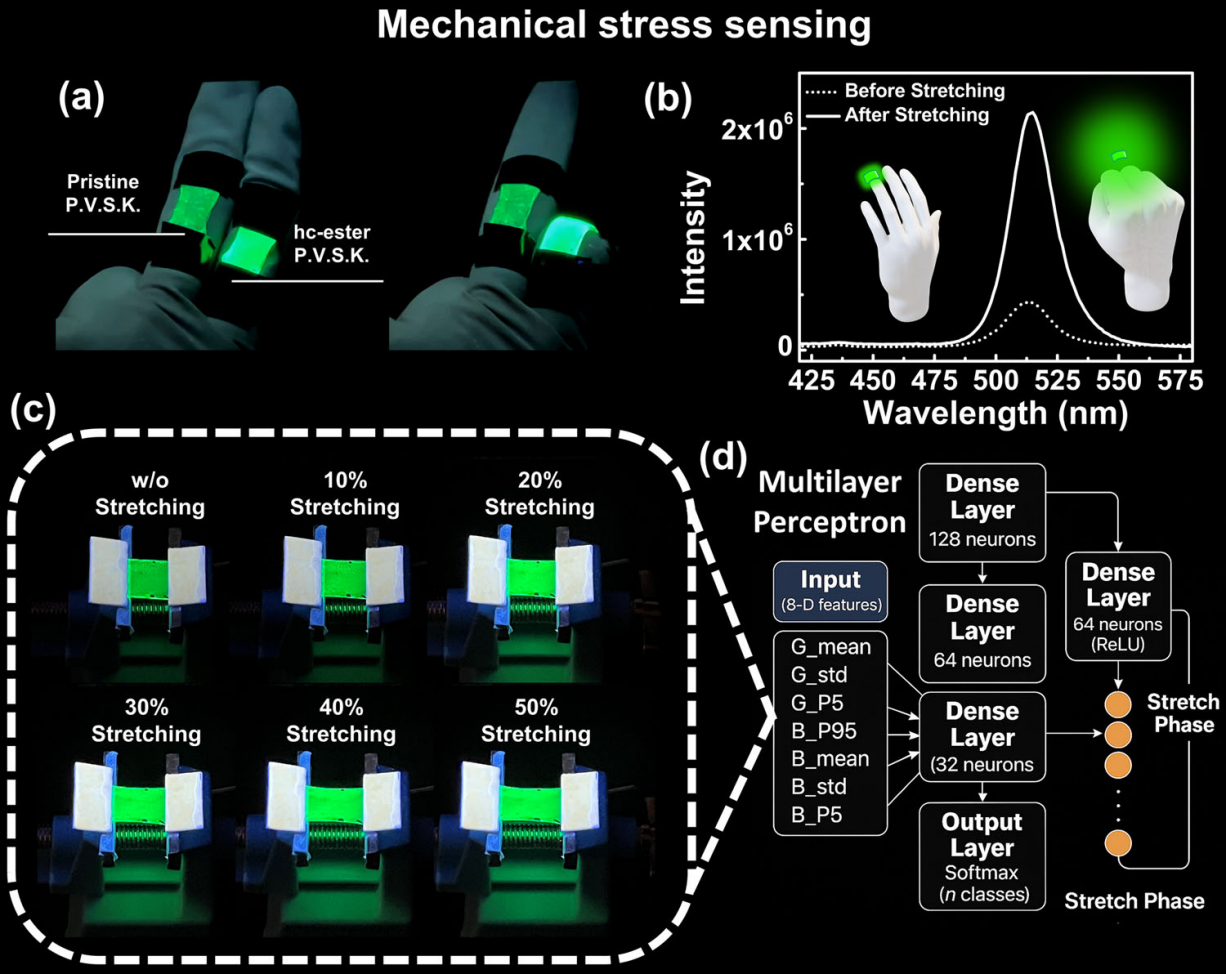
Application of ester‐based polymer‐perovskite composite in FMRD with logical emission signal for mechanical stress sensing. a) Photograph of a single‐finger‐mounted FMRD, where pristine P.V.S.K. and hc‐ester P.V.S.K. films are assembled on the proximal interphalangeal joints, respectively. b) PL spectra of hc‐ester P.V.S.K. before and after stretching. c) Emission behavior of the hc‐ester P.V.S.K. composite under different stretching ratios (0%–50%). d) Architecture of the multilayer perceptron model used for stretch‐phase classification, based on grayscale input feature, with successive dense layers and softmax output for signal recognition.

**Figure** **1** and **Figure** **2** illustrates the preparation of an FMRD with emission logic characteristics using perovskite synergized with a high‐crystallinity ester polymer (hc‐ester P.V.S.K.), applied in motion capture and mechanical stress sensing. The FMRD fabricated with P.V.S.K. facilitates longer‐range optical signal detection through its strong emission properties, eliminating the need for highly sensitive detectors to collect signals.

Within the motion capture application (Figure 1a), the hc‐ester P.V.S.K. is the emission layer for a flexible, bendable light‐emitting diode. This complex allows the FMRD to demonstrate varying brightness and electroluminescence spectra depending on the bending conditions, enabling the generation of dynamic logic signals that the motion capture system can recognize (Figure 1b and Video S1, Supporting Information). Figure S1 (Supporting Information) presents the performance measurements of the FMRD when unbent, achieving a maximum brightness of 643 cd/m^2^. Under different bending curvatures (Figure 1c), the FMRD operates at 4.5 V, exhibiting an increasing luminance trend with greater curvature (Figure 1d and **Table** **1**). Additionally, as shown in Figure S2 (Supporting Information), the external quantum efficiency (EQE) of the FMRD increases to 4.23% after bending, up from 3.4% in its unbent condition. The enhancement of EQE indicates that, under the same operating voltage conditions, a synergistic interaction between the hc‐ester and perovskite in the FMRD results in high energy transfer efficiency under strain conditions. Leveraging the luminance performance of the FMRD under different curvatures can serve as a signal source for varying motion intensities in motion capture applications. Additionally, the luminance difference of up to 30 cd/m^2^ under bending conditions, along with its self‐emission characteristics, enables the FMRD to act as a “point light source” with strong emission on moving parts. This enhances the precision of converting analog signals to digital ones. Figure 1e shows the results of cyclic bending tests on the FMRD under a voltage of 4.35 V and a bending curvature of 1.14 × 10^−2^ µm^−1^. Remarkably, the FMRD retains a clear brightness difference between unbent (0) and bent 1) states after twelve consecutive bending cycles, and the wavelength will not shift (Figure S3, Supporting Information). This continuous luminescence difference under continuous bending demonstrates the FMRD's ability to provide dynamic analog signal output for recognizing joint movements in motion capture systems. Excitingly, as shown in Figure 1f, the electroluminescence (EL) spectrum of the FMRD exhibits a 2 nm blue shift after bending (unbent: 516 nm; bent: 514 nm) along with a narrower full width at half maximum (FWHM) (unbent: 26.02 nm; bent: 23.22 nm). The distinct EL spectral performance before and after bending further enhances the signal discernibility and information accuracy of the FMRD in motion capture applications.

**Table 1:** The luminance performance of FMRD prepared with hc‐ester P.V.S.K. under different bending curvatures.

	Bending 1	Bending 2	Bending 3	Bending 4	Bending 5
Curvature	0.0 µm⁻¹	3.5 × 10⁻^3^ µm⁻¹	8.8 × 10⁻^3^ µm⁻¹	1.14 × 10^−2^ µm⁻¹	1.48 × 10^−2^ µm⁻¹
Luminance	96.22 cd m^2^	104 cd m^2^	119 cd m^2^	121 cd m^2^	128 cd m^2^

In the mechanical stress sensing application (Figure 2a and Video S2, Supporting Information), hc‐ester P.V.S.K.@FMRD and pristine P.V.S.K.@FMRD were assembled on the proximal interphalangeal joints of two different fingers. During the motion, the hc‐ester P.V.S.K.@FMRD exhibited enhanced photoluminescence intensity and a blue shift in emission wavelength in response to bending behavior. Figure S4 (Supporting Information) investigates the optical characteristics of hc‐ester P.V.S.K.@FMRD and pristine P.V.S.K.@FMRD before and after 10% stretching. First, in the unstretched condition, the photoluminescence (PL) emission intensity of hc‐ester P.V.S.K.@FMRD surpasses that of pristine P.V.S.K.@FMRD. Second, at excitation wavelengths of 390 nm and 450 nm, both FMRDs show a widening emission bandwidth. Significantly, with a 10% stretching strain, the emission continuity of FMRDs across a broad excitation wavelength spectrum improves. Additionally, under the same 10% stretching strain, the hc‐ester P.V.S.K.@FMRD of emission exhibits an intensity increase and no broadening of the bandwidth (Figure 2b). The differing emission continuity and emission bandwidth broadening under specific excitation energies before and after stretching enable the FMRD to generate distinct “light patterns” during the deformation process. This attribute enhances the FMRD's optical signal recognition capability, making the light signal discernible in mechanical stress sensing applications.

To realize the application of hc‐ester P.V.S.K.@FMRD in mechanical stress sensing, we first analyzed its photoluminescence behavior under various stretching conditions at a fixed distance of 1.2 cm using RGB grayscale image processing (Figure 2c; Figure S5, Supporting Information). By averaging the RGB grayscale values of pixels within this specified region (Figure S6, Supporting Information), we observed that the mean intensity of the green channel increased exponentially from 0% to 20% strain, reaching a saturation value of ≈250. Subsequently, within the 20%–60% strain range, the blue channel demonstrated a linear increase in intensity. Notably, the red channel remained consistently near zero throughout the stretching process, thereby serving as a stable baseline reference for the hc‐ester P.V.S.K.@FMRD recognition system. By incorporating the grayscale variation trends of the RGB channels under different strain conditions, along with brightness variation (standard deviation), the upper limit of the darkest 5% intensity (P5), and the lower limit of the brightest 5% intensity (P95), we successfully developed a stretch‐responsive photoluminescence recognition system for hc‐ester P.V.S.K.@FMRD, based on a multilayer perceptron architecture within a deep learning framework (Figure 2d and Supporting Code).

### 2.2 The Strain Effect of the Synergistic Interaction between the hc‐Ester and Perovskite

**Figure 3:**
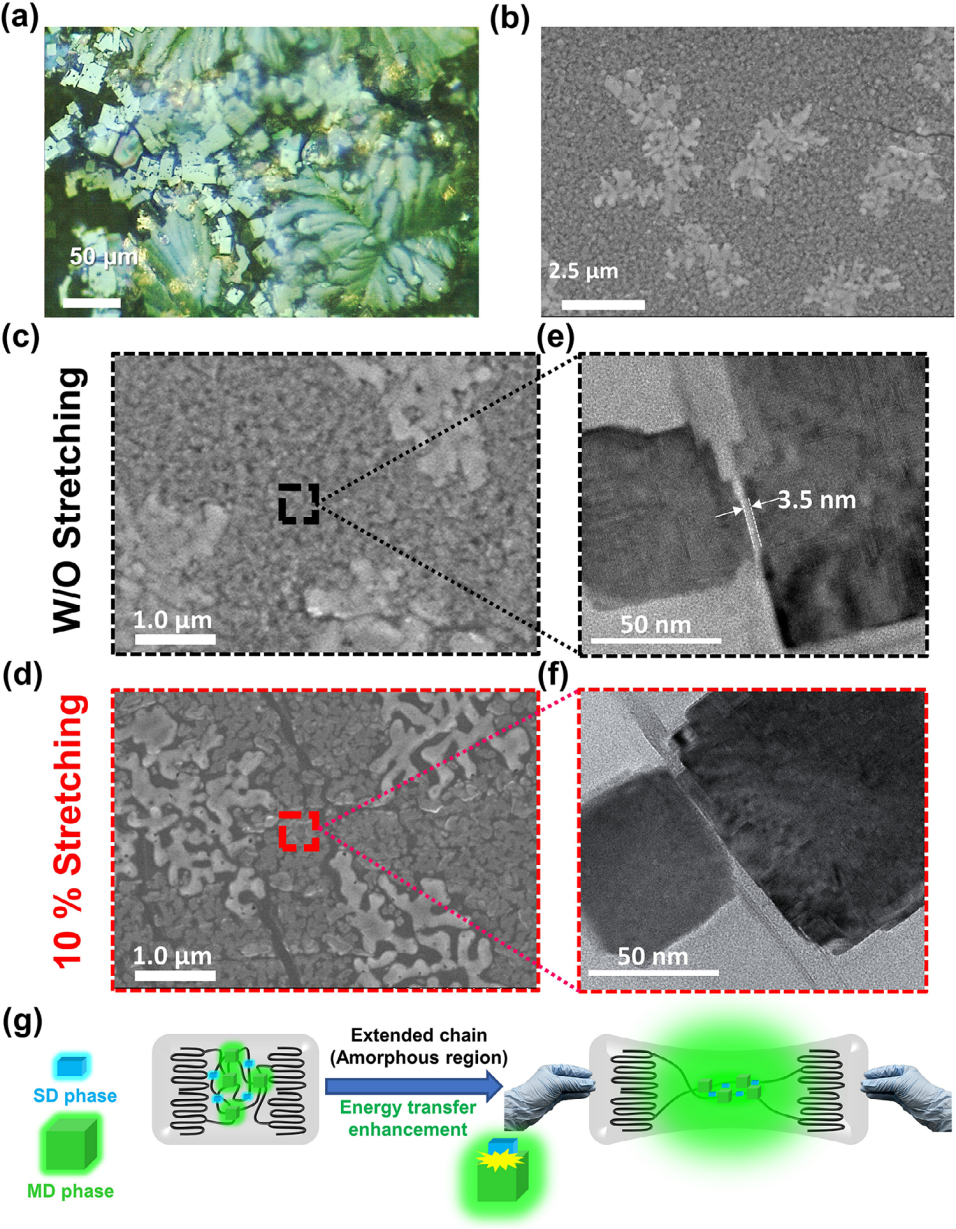
Application of ester‐based polymer‐perovskite composite in FMRD with logical emission signal. a) OM image of hc‐ester P.V.S.K. crystallized via natural deposition on PDMS. b,c) Under different magnification, SEM images of hc‐ester P.V.S.K. thermally annealed on PDMS before stretching; d) after stretching. e–f) TEM images of hc‐ester P.V.S.K. before and after stretching, respectively. g) Schematic illustration of chain extension in hc‐ester polymer inducing P.V.S.K. grain aggregation and facilitating energy transfer.

**Figure** **3** investigates the strain effect of the synergistic interaction between the hc‐ester and perovskite by comparing the morphology of hc‐ester P.V.S.K. grown on a flexible polydimethylsiloxane (PDMS) substrate before and after stretching. In Figure 3a, after hc‐ester P.V.S.K. undergoes crystallization through the natural deposition method with drop‐coating, the optical microscope (OM) visibly identifies distinct P.V.S.K. crystalline regions and the polymer spherulites. This crystallization behavior is similarly observed in the spin‐coating thermal annealing process, as depicted in Figure 3b, confirming that the crystallization behavior of hc‐ester P.V.S.K. remains unaffected by the choice of crystallization methods. According to the polymer extended chain behavior mechanism, ^[^ ^43^, ^44^, ^45^ ^]^ when a polymer is exposed to external forces from two directions (stretching or bending), the non‐crystalline regions of the chains become highly organized and aligned. Specifically, the surface morphology of PDMS/hc‐ester P.V.S.K. during stretching reveals a phenomenon of perovskite grain aggregation, which occurs along the ester chains due to the synergistic effect of polymer chain straightening, as found in scanning electron microscope (SEM) and transmission electron microscope (TEM) measurements (Figure 3c–f). Meanwhile, the folded spherulite regions of the hc‐ester chains maintain the structural properties of the hc‐ester P.V.S.K. during stretching movements. The P.V.S.K. used in this study consists of a mixture of low‐dimensional and high‐dimensional crystalline phases, exhibiting photoluminescence characteristics associated with both types of structures (as Figure 7b). As shown in Figure S7a,b (Supporting Information), further analysis of the crystal d‐spacing on either side of the ester chains confirms the presence of low‐dimensional (red) and high‐dimensional (blue) P.V.S.K. crystalline domains, respectively. Interestingly, due to the similar energy levels generated by low‐dimensional and high‐dimensional perovskite crystals, various forms of energy transfer can occur easily, enhancing the emission.^[^ ^46^, ^47^, ^48^, ^49^, ^50^ ^]^ The efficiency of energy transfer is determined by the spatial proximity between the two phases.^[^ ^49^ ^]^ Notably, Figure S8 (Supporting Information) time‐resolved photoluminescence spectroscopy (TRPL) measurements revealed that the photon lifetime of hc‐ester P.V.S.K. tends to increase under stretching conditions. This prolonged photon lifetime provides evidence that the energy transfer efficiency within the hc‐ester P.V.S.K. system is enhanced by mechanical deformation, thereby leading to improved photoluminescence intensity and conversion efficiency. hc‐ester P.V.S.K. The observed blue shift in the emission of hc‐ester P.V.S.K. under stretching is hypothesized to result from energy transfer from higher‐energy excitons, which promotes the population of higher‐energy excited states. This leads to a shift in the dominant emission toward higher photon energies. Given the above discussion, it can be inferred that the FMRD assembled by hc‐ester P.V.S.K. can utilize the chain straightening effect under external stretching and bending deformations to induce the aggregation of high‐ and low‐dimensional crystals in the P.V.S.K. This increases the probability of energy transfer, thereby improving luminescence and producing distinct spectral effects (Figure 3g).

### 2.3 Ion‐Dipole Interactions between Ester Polymer and Perovskite

**Figure 4:**
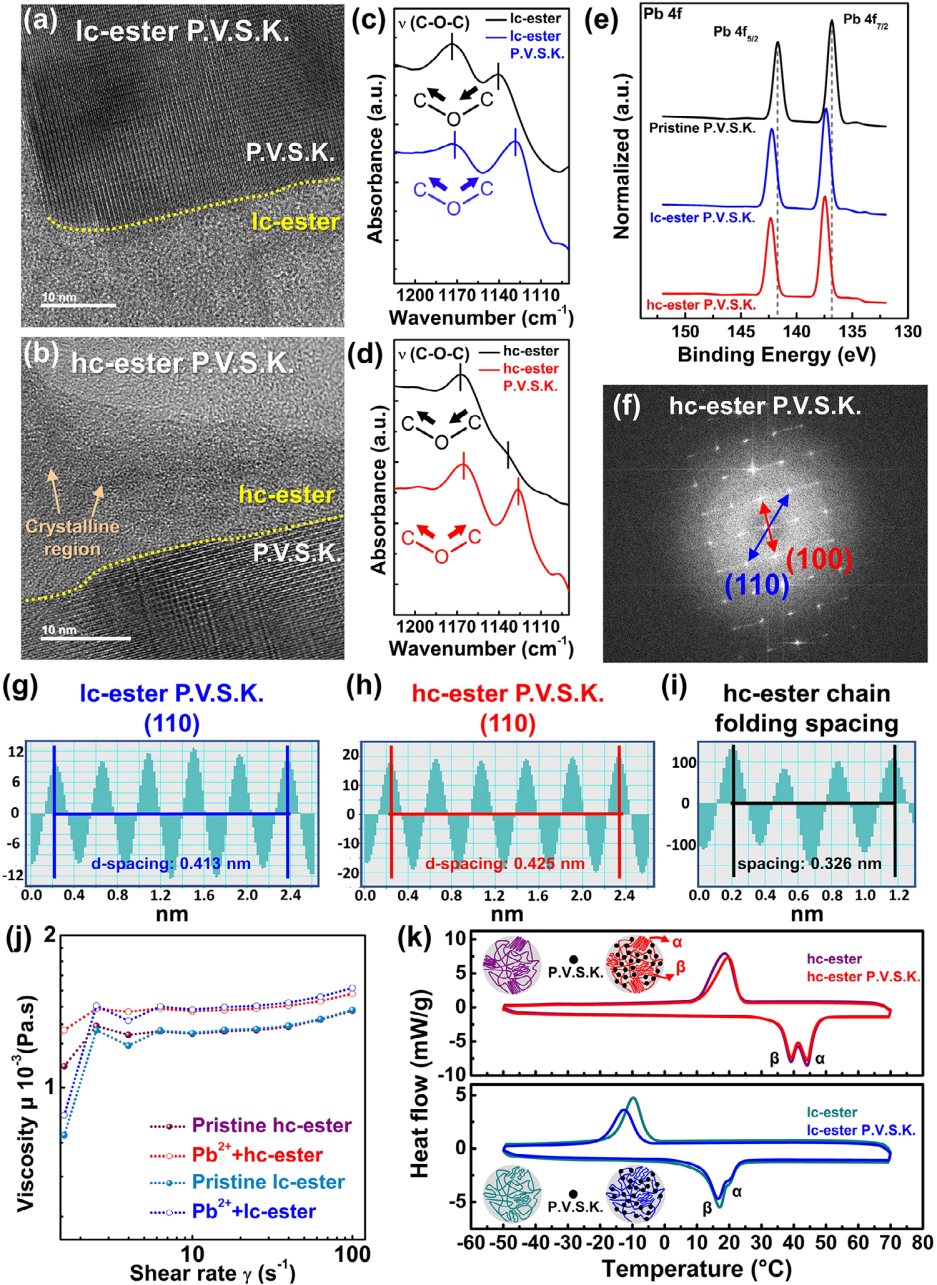
Polymer chain dynamic behavior after ion‐dipole interaction between crystalline ester‐based polymers and perovskite. TEM images of a) lc‐ester P.V.S.K. and b) hc‐ester P.V.S.K. νC‐O‐C stretching FTIR spectra of c) lc‐ester and d) hc‐ester. e) XPS spectrum of Pb 4f orbitals. f) FE‐TEM diffraction pattern of hc‐ester P.V.S.K. Lattice plane (110) d‐spacing diagram of g) lc‐ester P.V.S.K. and h) hc‐ester P.V.S.K. i) The d‐spacing diagram of hc‐ester crystallization region. j) Rheology plot of viscosity versus shear rate. k) DSC plot.

The crystallinity factor, which governs the physical properties of polymers, is believed to be influenced by polymer chain dynamics.^[^ ^51^ ^]^ However, current studies on blending polymers to regulate P.V.S.K. crystallization growth ^[^ ^36^, ^39^ ^]^ have not discussed the behavior of polymer chain folding crystallization following ion‐dipole interactions. Given this perspective, this study employs Poly(ε‐caprolactone) (PCL) as a high‐crystallinity ester polymer (hc‐ester) ^[^ ^52^ ^]^ and Poly(1,4‐butylene adipate) (PBA) as a low‐crystallinity ester polymer (lc‐ester) ^[^ ^53^ ^]^ to investigate whether ion‐dipole interactions between ester‐based polymers of different crystallinities and P.V.S.K. influence chain dynamics and system crystallization properties (**Figure** **4**). TEM observations (Figure 4a,b) reveal that blending ester polymers with P.V.S.K leads to the formation of a cross‐linked symbiotic composite structure through ion‐dipole interactions.^[^ ^35^ ^]^ Notably, inverse fast Fourier transform (FFT) TEM analysis confirms the presence of polymer chain folding crystallization regions in hc‐ester/P.V.S.K. blends, whereas no distinct polymer chain folding crystallization regions are observed in lc‐ester/P.V.S.K. blends (Figure S9, Supporting Information).

The ion‐dipole interaction between the ester polymer and P.V.S.K. can be confirmed using Fourier‐transform infrared spectroscopy (FTIR) and x‐ray photoelectron spectroscopy (XPS). This analysis focuses on the stretching vibration behavior of the ester group and the binding energy shifts of P.V.S.K. cations (Cs⁺, Pb^2^⁺). In an ion‐dipole interaction, the lone pair electrons on the oxygen atom of the ester group show high electron density, functioning as partial negatives (electron donors). On the other hand, the P.V.S.K. cations act as electron acceptors with partial positives.^[^ ^36^ ^]^

In Figure 4c,d, regardless of whether lc‐ester or hc‐ester is used, the addition of the P.V.S.K. precursor induces a shift in the C‐O‐C bond vibration mode from its asymmetric stretching to a lower‐energy symmetric stretching mode (lc‐ester: νC‐O‐C shifts from 1139.72 to 1128.15 cm^−1^; hc‐ester: νC‐O‐C shifts from 1133.46 to 1125.74 cm^−1^). Additionally, after incorporating the P.V.S.K. precursor into the ester polymer, the νC = O stretching signal broadens toward lower energy, indicating a transition toward more symmetric stretching behavior (Figures S10, S11, Supporting Information). The red‐shift of νC‐O‐C and νC = O to lower wavenumbers is possibly due to that the electron density distribution of the ester group becomes more delocalized after the addition of P.V.S.K. This indicates the formation of ion‐dipole interactions, where the ester group acts as a partial‐negative character. Furthermore, the enhanced symmetric stretching behavior of bothνC‐O‐C and νC = O implies that the interaction site is not restricted to a specific end of the C‐O‐C or C = O bonds. Instead, the ion‐dipole interaction is presumed to occur at the resonance site between C‐O‐C and C = O, where electron delocalization takes place.

XPS analysis was used to confirm the partial‐positive cation involved in the ion‐dipole interaction with the ester group. As shown in Figure 4e and Figure S12 (Supporting Information), the binding energy of the cations shifts toward higher energy after blending with the ester polymer, indicating that the cations act as electron acceptors in the ion‐dipole interaction. Notably, after adding the ester polymer, the binding energy of Pb^2^⁺ shifts by 0.50–0.70 eV to higher energy, whereas the binding energy of Cs⁺ shifts by only 0.35–0.55 eV to higher energy. The greater binding energy shift of Pb^2^⁺ suggests that the ion‐dipole interaction has more influence on it. This effect occurs because Pb^2^⁺ possesses more vacant orbitals available for electron hybridization than Cs⁺, leading to a stronger interaction with the ester group. The XPS results demonstrate that Pb^2^⁺ primarily governs the partial‐positive character in the ion‐dipole interaction. To further determine the specific interaction site between the ester polymer and Pb^2^⁺, the electron orbitals of the ester group were analyzed based on its structure (Figure S13, Supporting Information). In the ester group, the lone pair electrons on C‐O‐C are in an sp^3^ hybridized orbital, while those on C = O are in an sp^2^ hybridized orbital. As a result, the highest electron density in the ester group is located at the resonance site between C‐O‐C and C = O, where lone pair electron delocalization occurs. Additionally, the ester group's oxygen‐oxygen (O2–O1) distance is 2.261 Å, while the oxygen‐carbon (O2–C3) distance is 2.722 Å. This spatial resonance site between C‐O‐C and C = O provides a suitable coordination environment for accommodating the Pb^2^⁺ ion, which has an ionic radius of 1.19 Å.

Further investigation was conducted on the crystalline phase of P.V.S.K. after undergoing ion‐dipole interaction with the ester polymer. Diffraction pattern analysis (Figure 4f; Figure S14, Supporting Information), TEM inverse fast Fourier transform (FFT) analysis (Figure 4g,h), and X‐ray diffraction (XRD, Figure S15, Supporting Information) reveal that the crystal phase growth of P.V.S.K. remains unaffected by the addition of ester polymers. Moreover, under ion‐dipole interactions with ester polymers of different crystallinities, P.V.S.K. consistently exhibits a cubic crystal phase. Interestingly, upon examining the chain folding spacing of the ester polymer crystalline region and the P.V.S.K. (110) lattice plane d‐spacing in the hc‐ester P.V.S.K. case, it was found that the former is smaller than the latter (chain folding spacing: 0.326 nm; P.V.S.K. (110) d‐spacing: 0.425 nm) (Figure 4g–i). Additionally, in the XRD pattern of hc‐ester P.V.S.K., a crystalline signal associated with chain folding appears within the 20.5° to 22.5° range (Figure S16, Supporting Information). It can be inferred that the crystal phase crystallization of P.V.S.K. and the chain folding crystallization behavior of hc‐ester polymer are not affected by the ion‐dipole interaction between P.V.S.K. and the ester polymer.

The chain dynamic behavior of the ester polymer after ion‐dipole interaction was further investigated using rheological properties and differential scanning calorimetry (DSC) to verify that the polymer chain folding crystallization region remains unaffected by the interaction. In the rheological analysis (Figure 4j), it is observed that the viscosity of the ester polymer increases at the same shear rate after the introduction of P.V.S.K. According to the Stokes‐Einstein‐Sutherland equation (**Equation** **1**),^[^ ^54^ ^]^ the relationship between viscosity and the diffusion coefficient in a spherical particle system can be determined.

(1) $$D=\frac{k_{B}T}{6\pi \eta r}$$

D represents the diffusion coefficient, *k_B_* is the Boltzmann constant, *T* denotes the absolute temperature, η is the system viscosity, and *r* is the radius of the spherical particle. According to Equation (1), it can be inferred that Pb^2+^ cations, through ion‐dipole interactions, are adsorbed between ester polymer chains, reducing the overall diffusion energy of the solution, which in turn leads to an increase in viscosity. Interestingly, the viscosity increase of the Pb^2+^‐lc ester system is significantly higher than that of the Pb^2+^‐hc ester system. While a change of D does not necessarily translate polymer‐PVSK interaction, our FTIR result allowed us to decipher the diffusion mode of PVSK within the ester. Therefore, the higher viscosity observed in the Pb^2+^‐hc ester system can be attributed to the stronger ion–dipole interactions between Pb^2+^ ions and the functional groups of the hc‐ester polymer. These stronger interactions result in a more entangled or structured polymer–ion network, which increases resistance to flow and leads to more pronounced shear‐thinning behavior during rheological measurements. Conversely, the lower viscosity change in the Pb^2+^‐hc ester system suggests that the intermolecular forces responsible for chain folding crystallization in the hc‐ester may be stronger than the Pb^2+^ ester ion‐dipole interaction. As a result, the overall increase in solution viscosity is relatively lower. We use differential scanning calorimetry (DSC) results (Figure 4k) to understand the crystallization behavior of lc‐ester polymer and hc‐ester polymer exhibits different degrees of influence after the introduction of P.V.S.K. Adding P.V.S.K. shifts the lc‐ester polymer's crystallization temperature (T_c_) toward lower temperatures. The melting temperature (T_m_) of the low‐order chain folding crystalline phase (β‐crystalline phase) also shifts downward. The downward shift in T_c_ and T_m_ of lc‐ester P.V.S.K. after introducing P.V.S.K. indicates that lc‐ester is significantly affected by the strong ion‐dipole interactions formed due to its high concentration of amorphous regions. In contrast, the hc‐ester polymer, with its high concentration of chain‐folding crystalline regions, has a lower concentration of amorphous regions. This results in weaker ion‐dipole interactions that cannot impact the overall chain‐folding crystallization behavior.

It is hypothesized that the resistance of the chain‐folding crystalline region in hc‐ester polymer to ion‐dipole interactions arises from the additional intermolecular forces present in the structured crystalline region. According to the reference, beyond the typical intermolecular van der Waals forces, the ester group will generate additional intermolecular dipole‐dipole interaction ^[^ ^55^ ^]^ and intermolecular n→π* interaction ^[^ ^56^, ^57^ ^]^ between chains through C = O. Through the analysis of the relationship between chain dynamic behavior and ion‐dipole interactions confirms that in the hc‐ester polymer system with P.V.S.K., only the amorphous region engages in ion‐dipole interactions with Pb^2+^. In contrast, the ester groups within the chain‐folding crystalline region remain uninvolved in these interactions.

### 2.4 Crystal Dimension Growth and Surface Morphology of Ester Polymer‐Perovskite Composites

**Figure 5:**
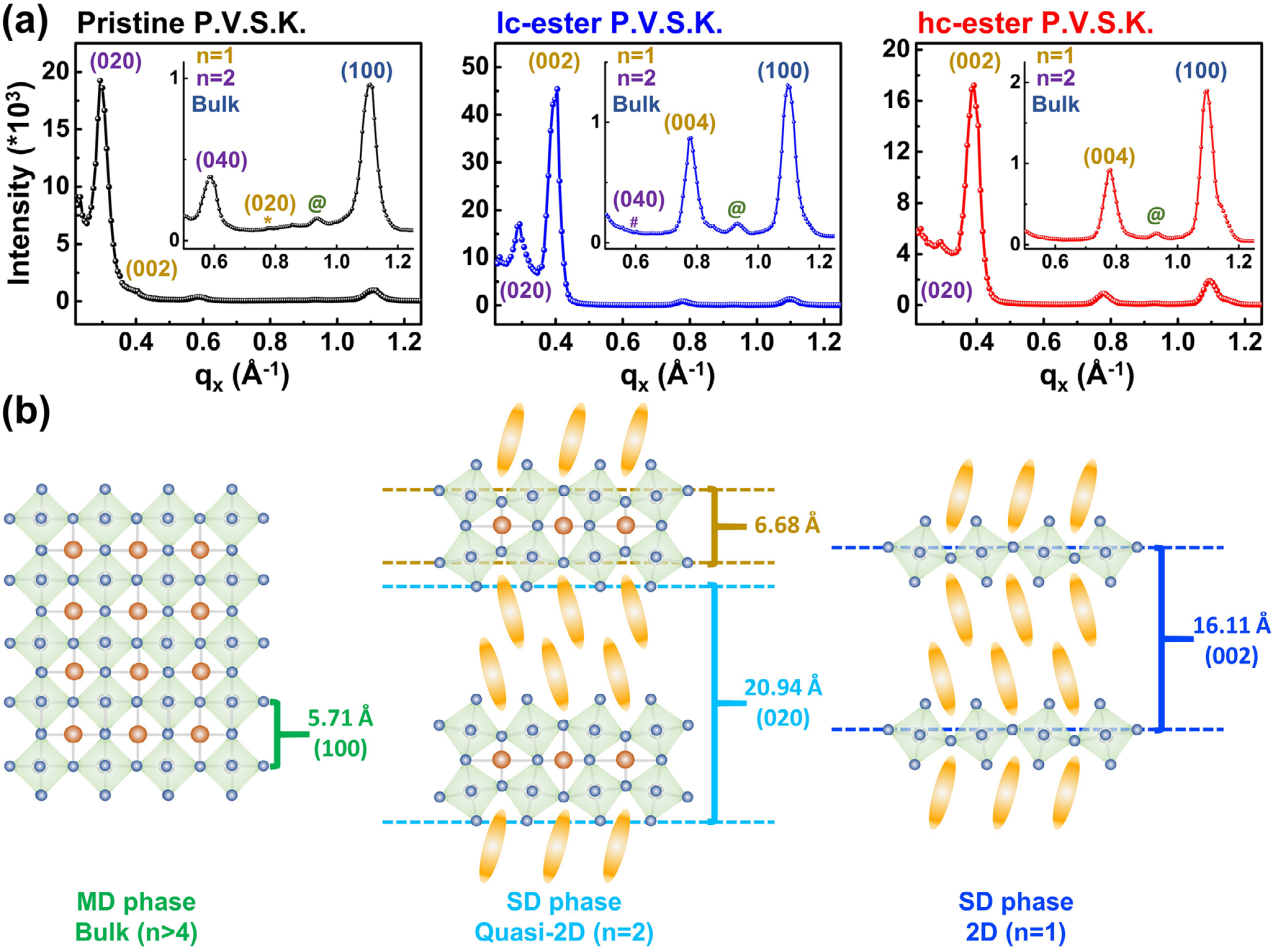
Analysis of the crystallization behavior of pristine P.V.S.K., lc‐ester P.V.S.K., and hc‐ester P.V.S.K. in the thin film state. a) GIWAXS 1D spectrum. b) Schematic diagram of MD (n>4) and SD (n = 1 to n = 4) crystal phases.

**Figure** **5** explores the crystal dimension growth and surface morphology of ester polymer‐perovskite composites. In the grazing‐incident wide‐angle x‐ray scattering (GIWAXS) measurements (Figure 5a; Figure S17, Supporting Information), it was found that the crystal dimensions of pristine P.V.S.K. were split into a main dimension (MD, n>4) and sub‐dimensions (SD, n = 1 and 4>n = 2) upon phenethylammonium (PEA) addition with high steric hindrance. Interestingly, after incorporating the ester polymer, the composition of the SD crystal dimension in P.V.S.K. showed a decreasing dimensionality trend, particularly observed in hc‐ester P.V.S.K., where the SD crystal composition shifted from being predominantly n = 2 (pristine P.V.S.K. case) to being dominated by n = 1 (Figure 5b). SD crystal restricted growth in hc‐ester P.V.S.K. is further evidenced by the gradual narrowing of the scattering signal within the q_z_ range of 0.175 to 0.325 Å^−1^ observed in 2D grazing‐incident small‐angle x‐ray scattering (GISAXS) measurements (Figure S18, Supporting Information). Notably, the coexistence of the chain‐folding crystalline regions of the hc‐ester and the P.V.S.K. crystals significantly enhances the structural heterogeneity within the hc‐ester P.V.S.K. system. This phenomenon is further validated by the 1D profile line cut analysis within the q_xy_ range of 0.05 to 0.1 Å^−1^ (Figure S19, Supporting Information), where a significant increase in the diffuse shoulder intensity of hc‐ester P.V.S.K. is observed, providing additional evidence of the system's elevated structural heterogeneity.

It is postulated that the SD crystal dimension of P.V.S.K. decreases after the addition of an ester polymer. This occurs because the ion‐dipole interaction between the ester group and P.V.S.K. produces “crystallized space confinement” during the crystallization process of P.V.S.K. The generation of crystallized space confinement can be explained by combining thermodynamics and kinetics. From a thermodynamic perspective, it is explained based on the nucleation rate equation (Equation 2) ^[^ ^58^ ^]^ established by the Arrhenius type equation.

(2) $$\frac{dN_{n}}{dt}=A\times exp\left(\frac{\Delta G^{*}}{k_{B}T}\right)$$

*N_n_* represents the number of nuclei, *A* is defined as a pre‐exponential factor related to temperature and molecular diffusion, ΔG* is the Gibbs free energy barrier for nucleation. During the ion‐dipole interaction process, the cation diffusion behavior of P.V.S.K. is restricted, which increases the Gibbs free energy barrier ΔG* formed by P.V.S.K. crystal nuclei and thereby reduces the nucleation rate $\frac{dN_{n}}{dt}$. Under conditions of reduced nucleation rate, P.V.S.K. crystallization behavior tends to concentrate at lower energy locations in confined spaces. Further, using the observed diffusion coefficient trend in the polymer‐P.V.S.K. solution, we explain the relationship between crystallized space confinement and the reduction of crystal dimension through crystallization kinetics‐Fick's first law (Equation 3).^[^ ^59^ ^]^

(3) $$J_{n}=4\pi r^{2}D\frac{dC}{dx}$$

Among them, J_*n*_, *r*, *D*, *C*, and *x*, respectively, represent the total flux of the crystal nucleus, the radius of the crystal nucleus, the diffusion coefficient, the concentration of the crystal nucleus at a specific distance (x), and the distance from the surface. The local concentration gradient induced by ion‐dipole interaction in the system restricts the migration of cations, reducing local diffusion flux J_*n*_ and reducing the growth rate of crystals in a confined space. This confinement effect further compresses the growth space of the crystal, resulting in more uniform growth of P.V.S.K. crystals and reduced size dimensions. Simultaneously, since the crystallization region of the hc ester does not undergo ion‐dipole interaction with P.V.S.K., it can create a more substantial crystallized space confinement effect at more specific spatial positions, leading to the formation of the lowest‐dimensional SD crystal composition.

**Figure 6:**
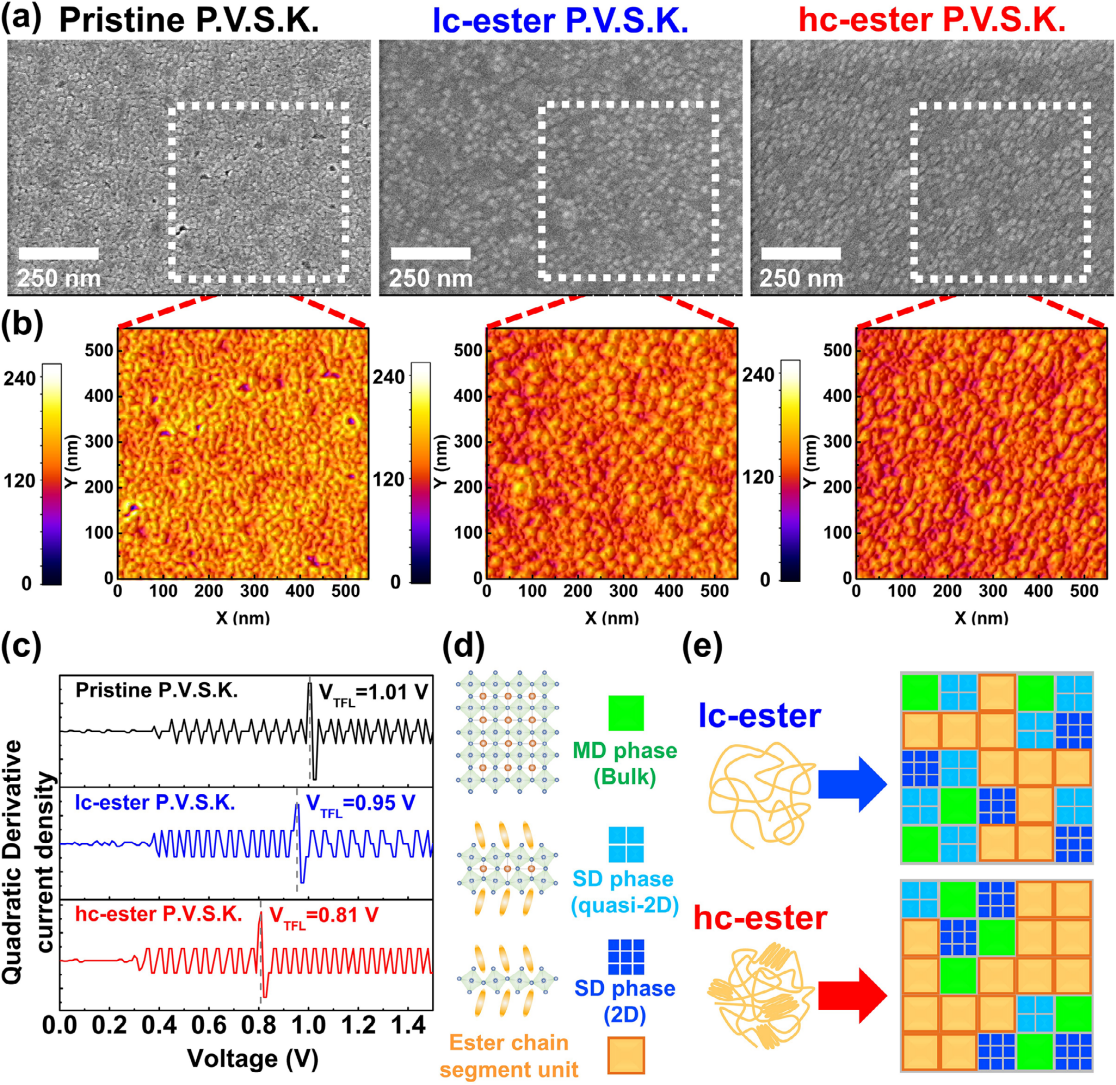
Analysis of the surface morphology of pristine P.V.S.K., lc‐ester P.V.S.K., and hc‐ester P.V.S.K. in the thin film state. a) SEM image and b) 3D image (analyzed and drawn through ImageJ software). c) Analyze the plot of electronic trap density using the J‐V characteristic curve. d) Schematic diagram illustrating how the hc‐ester polymer modulates the composition of P.V.S.K. crystal grain by adjusting the ratio of SD and MD phase domains.

**Figure** **6** examines the surface morphology of ester polymer‐perovskite composites. Based on the SEM observation results (Figure 6a,b), it is evident that the surface film of pristine P.V.S.K. shows multiple pinholes. The formation of these pinholes is possibly due to the wide range of crystal dimensions in the pristine P.V.S.K. system, leading to a stacking mismatch of the crystal grains.^[^ ^36^ ^]^ With the introduction of lc‐ester and hc‐ester polymers, the pinhole will be passivated through ion‐dipole interactions, enhancing the membrane's integrity. The extent to which P.V.S.K. forms pinholes can be quantified using measurements of electronic trap density.^[^ ^60^ ^]^ There is a positive correlation between electronic trap density and surface pinholes. When electrons pass through the surface pinhole of P.V.S.K., they become trapped and retained, resulting in a change of the original current transmission behavior from Ohm's law to Child's law, leading to the appearance of an inflection point known as the voltage trap‐filled limit (V_TFL_).^[^ ^61^ ^]^ Excessive pin holes are a negative factor that causes electrons to escape when P.V.S.K. is used in optoelectronic devices. In Figure 6c, hc‐ester P.V.S.K. shows the lowest V_TFL_ (pristine P.V.S.K.’s V_TFL_: 1.01 V; lc‐ester P.V.S.K.’s V_TFL_: 0.95 V; hc‐ester P.V.S.K.’s V_TFL_: 0.81 V), supporting the argument that hc‐ester can effectively inhibit surface pinhole formation. Additionally, the low electronic trap density results in the lowest operating electron escape rate when PeLEDs are applied. It must be noted that within the operating voltage range of 0 to 5.25 V (Figure S20, Supporting Information), the hc‐ester P.V.S.K. shows a nonlinear trend in current density increase. This indicates that the hc‐ester, as a polymer insulator, can function as a “carrier buffer,”^[^ ^36^ ^]^ helping to mitigate the damage caused by uncombined carriers to the P.V.S.K. layer.

Figure 6d,e presents a schematic diagram illustrating ester polymer‐P.V.S.K. composites. The addition of hc‐ester polymer enhances the crystallized space confinement factor for P.V.S.K. crystal growth, primarily driven by ion‐dipole interactions and the chain folding crystallization region. This confined growth reduces the SD crystal's dimensions and decreases the generation ratio, enlarging and homogenizing the P.V.S.K. crystalline grain domains. At the same time, the ester polymer preserves the crystalline nature of the chain folding.

### 2.5 Optical Properties and Exciton Dynamic of Ester‐Based Polymer‐Perovskite Composites

**Figure 7:**
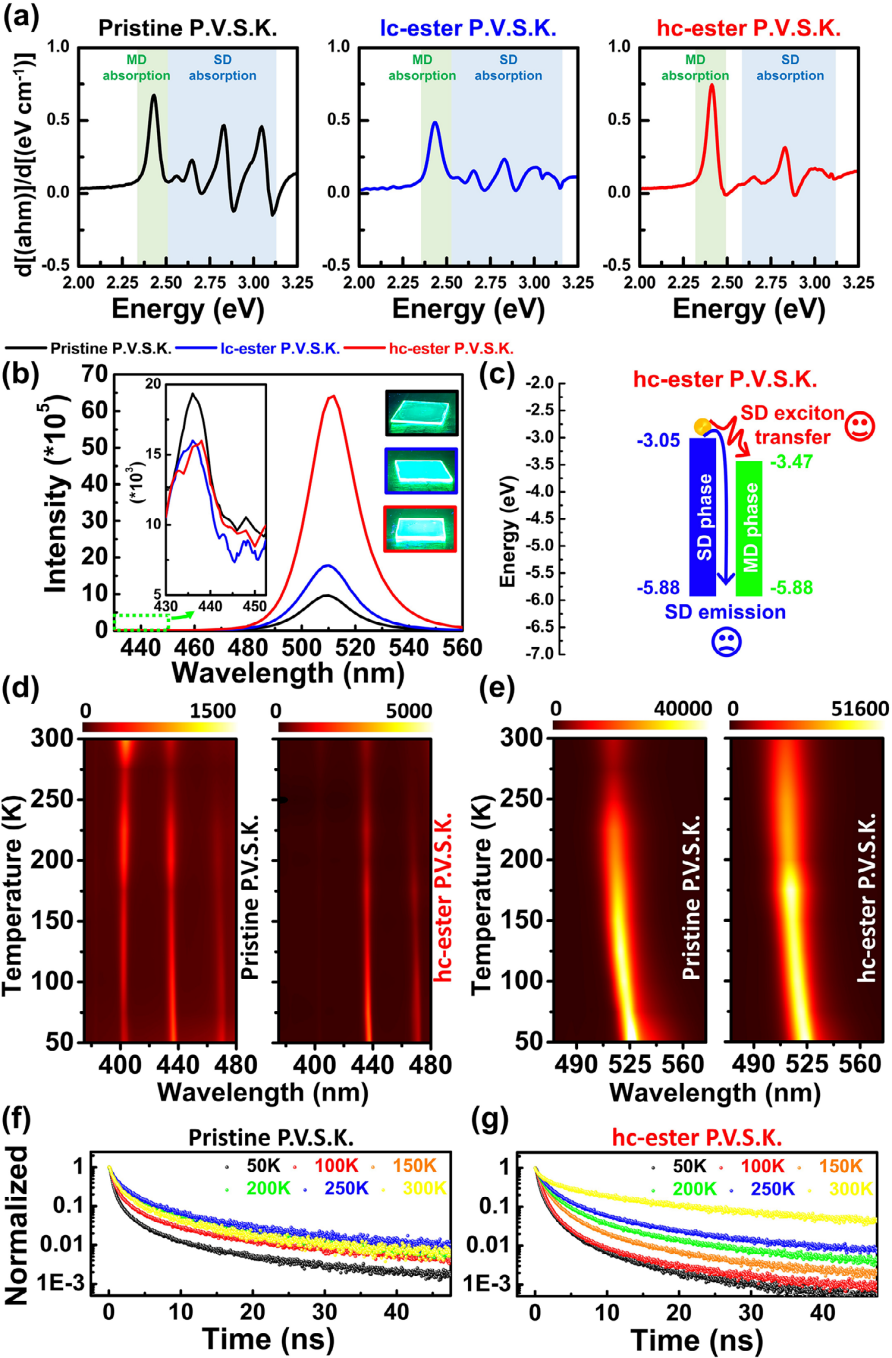
Optical characterization of ester‐based polymer–perovskite composites and exciton transfer behavior. a) First derivative spectra of Tauc plots showing the optical absorption characteristics of SD and MD phases in pristine, lc‐ester, and hc‐ester P.V.S.K. b) 1D‐PL spectra of the ester‐P.V.S.K. composites under 400 nm excitation, comparing the emission intensity change trend of SD and MD emission. c) Schematic illustration of SD‐to‐MD exciton transfer pathway in hc‐ester P.V.S.K. The 2D TD‐PL spectra of d) SD emission and e) MD emission from 300 to 50 K. g) TD‐TRPL of MD emission for f) pristine P.V.S.K. and g) hc‐ester P.V.S.K. at various temperatures.

**Figure** **7** further examines the optical properties of ester‐based polymer‐perovskite composites. By analyzing the UV‐Vis absorption spectrum (Figure S21, Supporting Information) along with the Tauc plot's first differential result (Figure 7a), we observe that P.V.S.K.’s SD and MD crystals have distinct absorption spectra. The SD absorption spectrum of pristine P.V.S.K. shows a multimodal broad distribution, whereas the SD absorption spectrum of P.V.S.K. blended with hc‐ester polymer displays a single‐peak narrow distribution. The multimodal and broad distribution of the SD absorption spectrum indicates that, in addition to 2D (n = 1) and quasi‐2D (n = 2), the SD crystal composition of pristine P.V.S.K. also includes higher‐dimensional quasi‐2D (n = 3 and n = 4) crystal phases. It is important to note that the SD absorption behavior resulting from crystallized space confinement by the hc ester polymer is primarily influenced by the narrow distribution of crystal phases in 2D (n = 1) and quasi‐2D (n = 2). Figure S22 (Supporting Information) illustrates the impact of the SD crystal's composition on the MD crystal's photoluminescence (PL) in the P.V.S.K. system, analyzed through 2D photoluminescence spectroscopy (2D‐PL). The SD crystal of pristine P.V.S.K. exhibits PL emission (SD emission) when the excitation wavelength (EX wavelength) coincides with the absorption wavelength range of the SD crystal (390 to 450 nm). Meanwhile, the emission from the MD crystal displays a broadening of the bandwidth across the EX wavelength range of 390 to 450 nm. It is speculated that the widespread distribution of crystal dimension composition in the pristine P.V.S.K. system is the primary cause of the broadening of the MD emission bandwidth. In particular, while the hc‐ester P.V.S.K. system also exhibits SD and MD emissions, the bandwidths of both are narrower and enhanced compared to the pristine P.V.S.K. system. Notably, Figure 7b shows that hc‐ester P.V.S.K. exhibits relatively weak SD emission and the most intense MD emission, suggesting that the narrow distribution of crystal dimensional composition in the hc‐ester P.V.S.K. system facilitates an energy transfer pathway from excitons generated in the SD phase to the excited states of the MD phase, thereby enhancing the MD emission (Figure 7c). According to previous research,^[^ ^48^ ^]^ when the perovskite of mixed MD/SD crystal is excited, the excitons in the SD excited state are readily induced by the lower energy MD excited state to form SD‐MD exciton transfer, enhancing the luminescence performance of MD emission. In addition, effective SD‐MD exciton transfer must overcome the inhomogeneous growth of the SD crystal.^[^ ^50^ ^]^

The dynamic behavior of excitons in the hc‐ester P.V.S.K. system was further investigated through temperature‐dependent photoluminescence (TD‐PL) and time‐resolved PL (TD‐TRPL) measurements. As shown in the TD‐PL spectra (Figure 7d,e), pristine P.V.S.K. exhibits nearly constant SD emission intensity from 300 to 50 K. In contrast, the MD emission becomes noticeably weaker between 300 and 250 K. This suggests that excitons in pristine P.V.S.K. predominantly prefer to return to the self‐excited state to generate SD emission. In contrast, the SD emission of hc‐ester P.V.S.K. displays a distinct temperature‐dependent behavior, where it increases as temperature decreases, and the MD emission remains strong and stable from 300 to 250 K. This trend indicates the presence of an efficient SD‐MD exciton transfer process within this temperature range. However, at temperatures below 200 K, the transfer pathway becomes suppressed, resulting in the accumulation of excitons in the SD phase and enhanced SD emission. Notably, at 50 K, pristine P.V.S.K. exhibits a pronounced asymmetry in the MD emission spectrum, attributed to exciton fine‐structure splitting caused by structural defects (Figures S24, S25, Supporting Information). Further analysis of the TD‐TRPL results (Figure 7f,g) shows that at room temperature, the MD emission lifetime of hc‐ester P.V.S.K. is longer than that of pristine P.V.S.K., indicating a delayed relaxation process. This delay is attributed to energy transfer from higher‐energy SD excitons to lower‐energy MD excited states.^[^ ^46^ ^]^ Consistent with the TD‐PL observations, the MD emission lifetime of hc‐ester P.V.S.K. decreases progressively with lowering temperature, suggesting that the SD‐MD exciton transfer process becomes increasingly hindered under cryogenic conditions. The above observations suggest that the limited exciton transfer efficiency in pristine P.V.S.K. originates from its broad distribution of crystal dimensional compositions, which leads SD excitons to favor radiative recombination within the SD phase. In contrast, the narrower dimensional distribution induced by the hc‐ester polymer facilitates more effective exciton transfer from the SD to MD excited states, thereby enhancing the MD emission intensity. As a result, when hc‐ester P.V.S.K. is integrated into a rigid planar PeLED device, the optimized energy transfer behavior enables superior green emission performance, achieving a peak luminance of 13 250 cd m^2^ (Figure S26, Supporting Information), a narrow full‐width at half‐maximum of 19.77 nm (Figure S27, Supporting Information), and a suppressed EQE roll‐off (Figure S28, Supporting Information).

## 3 Conclusion

This study successfully developed an FMRD with optical logic emission properties by utilizing ion‐dipole interactions in ester‐based polymer‐perovskite composites. The high‐crystallinity ester polymer regulates sub‐dimensional perovskite crystal growth through “crystallized space confinement,” thereby enhancing exciton transfer efficiency and luminescence performance. Furthermore, the high‐crystallinity ester polymer improves the surface morphology of perovskite films and reduces electron trap density, showcasing the strong potential for optoelectronic applications. The FMRD displays dynamic luminescence changes under mechanical deformation, making it highly suitable for motion capture and mechanical stress sensing. These findings offer new insights into integrating crystallization engineering with perovskite‐based optoelectronics to explore multi‐spectral optical logic applications for next‐generation flexible optoelectronic devices.

## Conflict of Interest

The authors declare no conflict of interest.

## Supporting information



Supporting Information

Supplemental Video 1

Supplemental Video 2

Supporting Information

## Data Availability

The data that support the findings of this study are available from the corresponding author upon reasonable request.
