# Supplementary material for: Flexible Mechanical Response Device With Optical Logic Emission Enabled by Synergistic Crystallization Engineering of Ester Polymer and Perovskite
Source: Adv Sci (Weinh). 2025 Aug 13;12(43):e08812. doi: 10.1002/advs.202508812 (PMC12631842; doi:10.1002/advs.202508812)
Supplement: Supplementary file 1 — Supporting Information [file ADVS-12-e08812-s002.docx]

***Supplementary information***

**Flexible Mechanical Response Device with Optical Logic Emission Enabled by Synergistic Crystallization Engineering of Ester Polymer and Perovskite**

Zhen-Li Yan ^a,b^, Tzu-Ming Hsu ^b^, Chien-Hsin Wu ^a,c^, Jean-Sebastien Benas ^b^, Ying-Chi Huang ^a^, Wei-Cheng Chen ^d^, Bi-Hsuan Lin ^e^, Mei-Hsin Chen ^f^, Ja-Hon Lin ^f^, Hsinhan Tsai ^g,h^, Chu-Chen Chueh ^d^, Chihaya Adachi ^I,^*, Ru-Jong Jeng ^a,^*, and Chi-Ching Kuo ^b,^*

^a^ Institute of Polymer Science and Engineering, Advanced Research Center for Green Materials Science and Technology, National Taiwan University, Taipei 10617, Taiwan

^b^ Institute of Organic and Polymeric Materials, National Taipei University of Technology, Taipei 10608, Taiwan

^c^ Advanced Research Center for Green Materials Science and Technology, National Taiwan University, Taipei 10617, Taiwan

^d^ Department of Chemical Engineering, National Taiwan University, Taipei 10617, Taiwan

^e^ National Synchrotron Radiation Research Center, 101 Hsin-Ann Road, Hsinchu Science Park, Hsinchu 30076, Taiwan

^f^ Department of Electro-Optical Engineering, National Taipei University of Technology, Taipei 10608, Taiwan

^g^ Department of Chemistry, University of California, Berkeley, CA, USA

^h^ Department of Physics, SUNY University at Buffalo, Buffalo, NY, USA

^I^ Department of Applied Chemistry, Kyushu University, 744 Motooka, Nishi, Fukuoka 819-0395, Japan

^*^Author to whom all correspondence should be addressed

Tel.: 886-2-27712171*2407

Correspondence Author: Prof. C. Adachi (E-mail: adachi@cstf.kyushu-u.ac.jp)

Prof. R.-J. Jeng (E-mail: rujong@ntu.edu.tw)

Prof. C.-C. Kuo (E-mail: kuocc@mail.ntut.edu.tw)

**Experimental**

**Materials**

Poly(butylene adipate) (PBA M.W. 2000) (Tc:-9.47℃ from **Figure 4k**) and polycaprolactone (PCL M.W. 2000) (Tc:18.26℃ from **Figure 4k**) were purchased from DAICEL Corp. Lead(II) bromide (PbBr_2_, 99%), phenethylammonium (PEABr, >98%), cesium bromide (CsBr, >99.9%), dimethyl sulfoxide (DMSO, 99.9%), poly(9-vinylcarbazole) (PVK, average Mn 25,000-50,000), chlorobenzene (CB, 99.8%), 2 ,2',2''-(1,3,5-benzinetriyl)-tris(1-phenyl-1-H-benzimidazole) (TPBi, 99%), lithium fluoride (LiF, ≥99%) were purchased from Sigma-Aldrich. Aluminum (Al) was provided by Summit-Tech Co., and patterned indium tin oxide (ITO) substrates with dimensions of 30 × 30 × 0.7 mm³ and surface resistance of 5 Ω were purchased from Lumtec Ltd. Agent A and agent B of polydimethylsiloxane (PDMS) were purchased from Krayden Dow Sylgard 184 Silicone Elastomer Kit.

**Perovskite precursor solutions preparation**

Mix 19.68 mg (0.1 mmol) of phenethylammonium bromide (PEABr) and 49.8 mg (0.23 mmol) of cesium bromide (CsBr) with 71.55 mg (0.2 mmol) of lead (II) bromide (PbBr_2_) in 1.5 ml of dimethyl sulfoxide (DMSO), then stir overnight at 50°C. Add 1 ml of PEA-CsPbBr3 precursor to either 3.0 mg of polycaprolactone (PCL-hc-ester; M.W. 2000) or 3.0 mg of poly(1,4-butylene adipate) (PES-lc-ester; M.W. 2000), and dissolve with stirring at 50°C for 30 minutes.

**Perovskite thin film fabrication**

The quartz glass substrate was sonicated with deionized water, acetone, and isopropanol for 15 minutes, followed by oxygen plasma treatment for 5 minutes. The perovskite precursor was spin-coated at 3000 rpm for 90 s on the bare quartz glass, then annealed at 80 ◦C for 15 min. All perovskite thin film spin-coating processes were performed at room temperature in an N_2_-filled glove box.

**Perovskite trap density device fabrication**

Patterned ITO indium-tin oxide-coated glass substrates were sonicated with deionized water, acetone, and isopropanol for 15 min, followed by oxygen plasma treatment for 5 min. The perovskite precursor was spin-coated at 3000 rpm for 60 s on the ITO glass, then annealed at 80 ◦C for 15 min. At last Al (50 nm) were deposited by thermal evaporation under a high vacuum (~10-6 Pa) environment. All perovskite thin film spin-coating processes were performed at room temperature in an N_2_-filled glove box.

**PeLEDs device (ITO glass substrate) fabrication**

Patterned ITO indium-tin oxide-coated glass substrates were sonicated with deionized water, acetone, and isopropanol for 15 min, followed by oxygen plasma treatment for 5 min. Firstly, use the graphene quantum dot-PEDOT:PSS composite developed by Yan et al.**^[34]^** was used at 3000 rpm for 45 seconds and annealed at 120℃ for 15 min. Subsequently, the PVK precursor solution (12 mg/2 ml in chlorobenzene) was filtered using a 0.45 μm PVDF filter and spin-coated onto the ITO glass at 3000 rpm for 45 seconds and annealed at 150°C for 15 minutes. Next, the EML (emission layer) perovskite precursor solution was spin-coated at 3000 rpm for 90 seconds and annealed at 80°C for 15 minutes. At last, TPBi (35 nm), LiF (1 nm), and Al (90 nm) were deposited by thermal evaporation under a high vacuum (~10^-6^ Pa) environment. The PeLEDs with an active area of 4 mm^2^ were encapsulated using epoxy glue and glass covers. HTL and EML thin film deposition processes were completed in an N_2_-filled glove box.

**FMRD fabrication**

**PET substrate (for bending)**

Patterned ITO indium-tin oxide-coated PET substrates were sonicated with deionized water, acetone, and isopropanol for 15 min, followed by oxygen plasma treatment for 5 min. Firstly, use the graphene quantum dot-PEDOT:PSS composite developed by Yan et al.**^[34]^** was used at 3000 rpm for 45 seconds and annealed at 120℃ for 15 min. Subsequently, the PVK precursor solution (12 mg/2 ml in chlorobenzene) was filtered using a 0.45 μm PVDF filter and spin-coated onto the ITO PET at 3000 rpm for 45 seconds and annealed at 150°C for 15 minutes (thickness: 15 nm). Next, the EML (emission layer) perovskite precursor solution was spin-coated at 3000 rpm for 90 seconds and annealed at 80°C for 15 minutes (thickness: 40 nm). At last, TPBi (35 nm), LiF (1 nm), and Al (90 nm) were deposited by thermal evaporation under a high vacuum (~10^-6^ Pa) environment. The PeLEDs with an active area of 4 mm^2^ were encapsulated using polyethylene hot press sealing technology. HTL and EML thin film deposition processes were completed in an N_2_-filled glove box.

**PDMS substrate (for stretching)**

Mix Agent A and Agent B of PDMS precursor in a weight ratio of 10:1, then stir for 5 minutes. Place the PDMS solution in the refrigerator to defoam for 2 hours. The PET substrates were sonicated in deionized water, acetone, and isopropyl alcohol for 15 minutes, followed by treatment with oxygen plasma for 30 seconds. Drop 1 ml of PDMS solution onto a 5x5 cm² PET substrate and spin-coat at 350 rpm for 45 seconds. Finally, perform annealing at 80°C for 2.5 hours and peel off the PDMS film.

The PDMS film was adsorbed onto the PET substrate and treated ultrasonically with isopropyl alcohol for 15 minutes, followed by oxygen plasma for 2 minutes. Finally, the hc-ester/lc-ester perovskite precursor was spin-coated at 3000 rpm for 60 seconds and annealed at 80 °C for 15 minutes. (Repeat this step twice)

**Development of the Stretch Ratio Prediction Program Based on RGB Gray Value Analysis**

To investigate the photoluminescent response of ester polymer–perovskite composites under mechanical stretching, we developed an interactive MATLAB tool capable of predicting the stretch ratio by analyzing statistical parameters derived from RGB grayscale values. In this model, the red channel serves as a reference baseline and is excluded from analysis.

**Construction of the RGB Gray Value Database**

A total of 13 sets of photoluminescence images were acquired under stretching conditions ranging from 0% to 60%. ImageJ was used to extract pixel gray values from the green and blue channels. For each set, four statistical features were calculated:

- Mean
- Standard Deviation (Std)
- 5th Percentile (P5%)
- 95th Percentile (P95%)

These statistics form an 8-dimensional input vector:

$$[G_{mean},G_{std},G_{P5},G_{P95},B_{mean},B_{std},B_{P5},B_{P95}]$$

The corresponding stretch ratio serves as the model’s target output.

All data is hard-coded directly within the MATLAB script for maximum efficiency and independence from external files:

*“predictors = [...]; % 8-dimensional green/blue feature vectors*

*responses = [...]; % Actual stretch ratios”*

**Model Construction Using Gaussian Process Regression (GPR)**

To capture the nonlinear relationship between RGB-derived features and mechanical strain, we applied a “Gaussian Process Regression (GPR)” model:

*“model = fitrgp(predictors, responses);”*

GPR is selected due to its robustness with small datasets and its superior interpolation performance. Once trained, the model can predict stretch ratios from unseen input data with high flexibility.

**Graphical User Interface for Interactive Input**

A user-friendly input dialog allows direct entry of the statistical RGB values. The interface requires four entries (G­_mean_, G_std_, B_mean_, B_std_):

*“prompt = { 'G Mean:', 'G Std:', 'B Mean:', 'B Std:' };*

*answer = inputdlg(prompt, dlg_title, num_lines, defaultans);”*

Upon valid input, the model outputs the predicted stretch ratio in real-time:.

*“pred_ratio = predict(model, x);*

*msgbox(sprintf('✅ Predicted Stretch Ratio: %.3f (%.1f%%)', pred_ratio, pred_ratio * 100), 'Prediction Result');”*

This immediate feedback allows for rapid screening and classification of photonic stretching states.

**System Applicability and Extensibility**

The developed MATLAB tool offers a streamlined and non-image-based approach to estimating mechanical strain from photoluminescent data. The system is also scalable. Additional training data can enhance prediction accuracy, and the regression model can be swapped for more advanced techniques such as decision trees or neural networks to accommodate complex or nonlinear trends.

**Analysis and Characterization**

**TEM analysis**

10 μl of the mixed precursor liquid of ester-based polymer and perovskite was dropped on the carbon-copper grid and dried overnight in a vacuum. Image recording and measurements were performed using a field emission transmission electron microscope (FE-TEM, model JEOL JEM-2100F, Instrument Center, National Taiwan University). D-spacing was analyzed with a Digital Micrograph.

**Rheological analysis**

Take 15 ml of the mixed precursor solution of ester-based polymer, lead bromide, and place it on the cup-shaped rheological stage. Rheological measurements (2 rounds at 50°C flow mode; shear rate 0.1~300 s-1) were performed using an AR2000ex system (stress control), TA Instruments, Institute of Polymer Science and Engineering, National Taiwan University).

**DSC analysis**

Drop 1 ml of the mixed precursor solution of ester-based polymer and perovskite into the Teflon stage, dry it under vacuum at 80°C for 4 days, and collect the sample. Take a 4.5 mg sample for DSC measurement; use TA Instruments Q-20, Institute of Polymer Science and Engineering, National Taiwan University) for DSC measurement (measurement temperature range: -50℃~70℃; temperature rising and cooling rate: 10℃/min; 3 rounds).

**XRD analysis**

150 μl of the mixed precursor solution of ester-based polymer and perovskite was spin-coated on the full-version ITO substrate, and XRD patterns (Angle range: 10°~50°) were obtained through PANalytical diffractometer (X’ Pert3 Powder).

**GIWAXS analysis**

150 μl of the mixed precursor solution of ester-based polymer and perovskite was spin-coated on the full-version ITO substrate, and GIWAXS patterns (Angle range: 0.12-0.2; Exposure time: 1s) were obtained on Beamline 7.3.3 of Advanced Light Source (ALS), Lawrence Berkeley National Laboratory.

**GISAXS analysis**

150 μl of the mixed precursor solution of ester-based polymer and perovskite was spin-coated on the full-version ITO substrate, and GISAXS patterns were obtained at the BL23A (TLS) beamline of the National Synchrotron Radiation Research Center (NSRRC) in Taiwan.

**Optical analysis**

150 μl of the mixed precursor solution of ester-based polymer and perovskite spin-coat it on the quartz glass substrate, then perform the following optical analysis.

**UV–visible absorption spectrum**

Measure the UV–vis absorption spectra of perovskite films using a Jasco V-730 spectrometer.

**Photoluminescence (PL&2D-PL) spectrum**

The PL and 2D-PL spectra of perovskite were measured using a dynamic fluorescence excitation spectrometer (Nanolog, Horiba Scientific).

**Temperature-dependent of photoluminescence (TD-PL) and time-resolved photoluminescence spectroscopy (TD-TRPL)**

Measurements were performed using a spectrometer (model iHR320, HORIBA) combined with a Hamamatsu C10910 streak camera. The excitation light source was a PLP-10 laser diode head (M10306-27) with an M10913 slow single scan unit. A cryogenic control system based on compressed helium flow was employed to regulate low-temperature conditions. All measurements were conducted at the National Synchrotron Radiation Research Center (NSRRC, Taiwan).

**SEM analysis**

Take 150 μl of the mixed precursor solution of ester-based polymer and perovskite spin-coat it on the quartz glass substrate, and conduct SEM analysis using a field emission scanning electron microscope (FE-SEM, model Hitachi S4800, 10 kV acceleration voltage) (use imageJ software for image analysis).

**Trap density analysis**

Measurement of trap density in voltage-varying mode with a 0.01V interval from 0 to 2V.

**Optical microscope analysis**

1 ml of the mixed precursor solution of ester-based polymer and perovskite was dropped onto quartz glass, vacuum dried at 50°C for 2 days, and optical microscopic measurement was performed using SAGE Vision HM-3006.

**PeLEDs and FMRD performance: current density-voltage (IV) and luminance-voltage (LV) characteristics**

Measured using Keithley 2400 and SpectraScan PR670 photon multichannel analyzers.

**PeLEDs and FMRD performance: EL spectrum**

Electroluminescence spectra were collected using a SpectraScan PR670 photon multichannel analyzer.

**Statistical Analysis**

1. **Data Visualization and Software**

All statistical figures and numerical plots were generated using OriginPro 8.5. Grayscale statistics were processed in ImageJ 1.54h, and regression modeling was implemented in MATLAB R2023b (Statistics and Machine Learning Toolbox). All normalized analysis figures are scaled from 0 to 1 for comparison.

1. **Photoluminescence Gray Value Analysis**

For RGB-based photoluminescence (PL) image analysis, grayscale profiles were extracted using ImageJ (v1.54h) along a defined 1.2 cm axis on the stretched films. Only green and blue channels were analyzed as active predictors, with the red channel used as a passive reference baseline. Each line profile contained over 500 pixel values. Outlier removal was performed using interquartile range (IQR) filtering. From each channel, four statistical features were calculated: mean, standard deviation (SD), 5th percentile (P5%), and 95th percentile (P95%). These features were summarized as means and plotted using OriginPro 8.5.

1. **Stretch Ratio Modeling from RGB Statistics**

A regression model was trained in MATLAB R2024a using built-in RGB statistical features extracted from PL images at different stretch ratios (n = 13, from 0% to 60%). The model utilized Gaussian Process Regression (GPR) via the *_fitrgp()_* function, which took an 8-dimensional input vector composed of G/B channel statistics. No data transformation was applied. The stretch ratio prediction performance was evaluated using the root-mean-square error (RMSE) and residual inspection. No classical hypothesis testing or alpha adjustment was performed. All training and testing datasets were embedded internally within the model script.


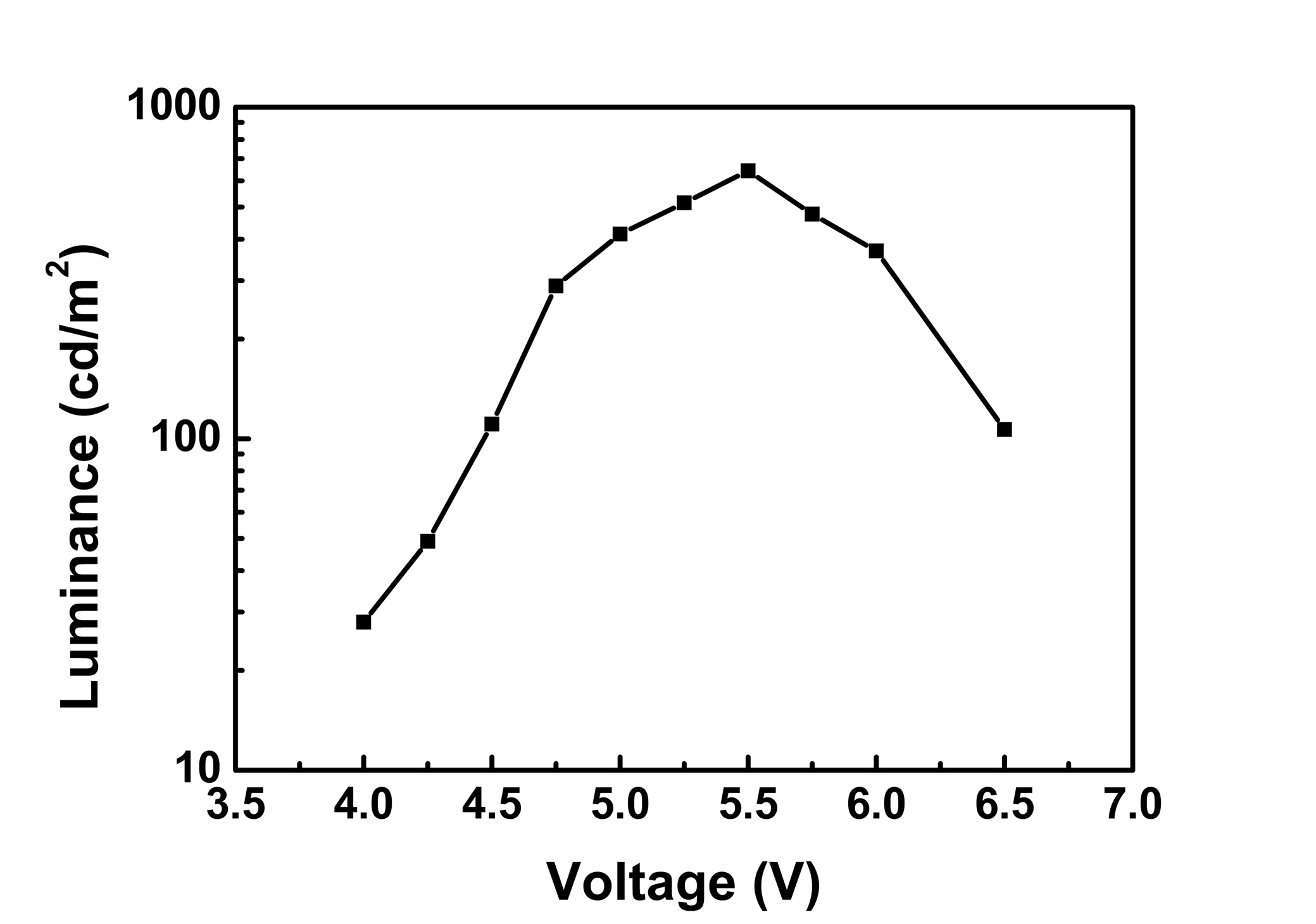


**Fig. S1**. The luminance performance of the flexible light-emitting diode-EL-FMRD made with hc-ester P.V.S.K. under varying voltage operation.


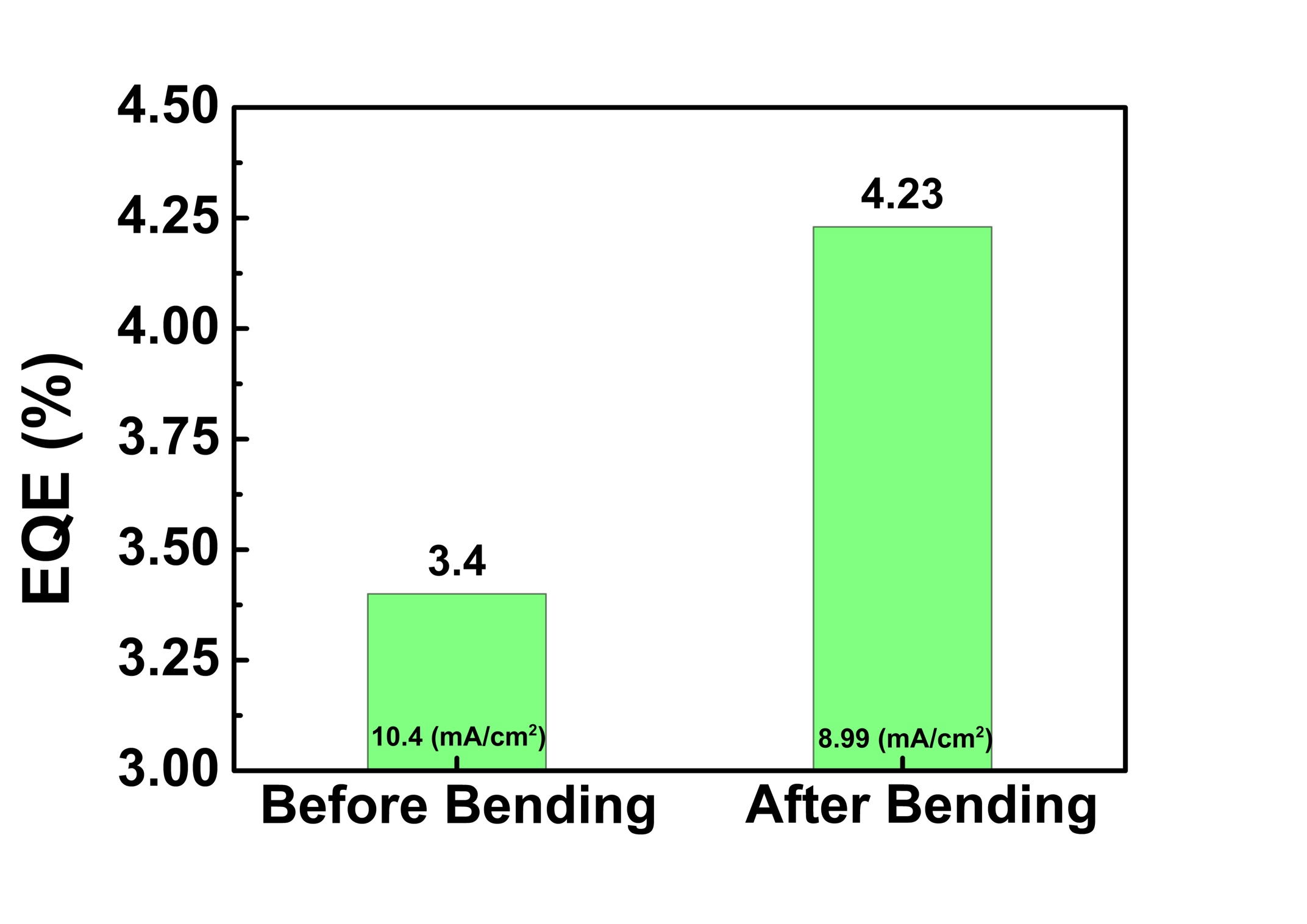


**Fig. S2**. Compare the EQE performance of EL-FMRD before and after bending at 5.0 V operation.





**Fig. S3**. EL stability of FMRD under cyclic bending.


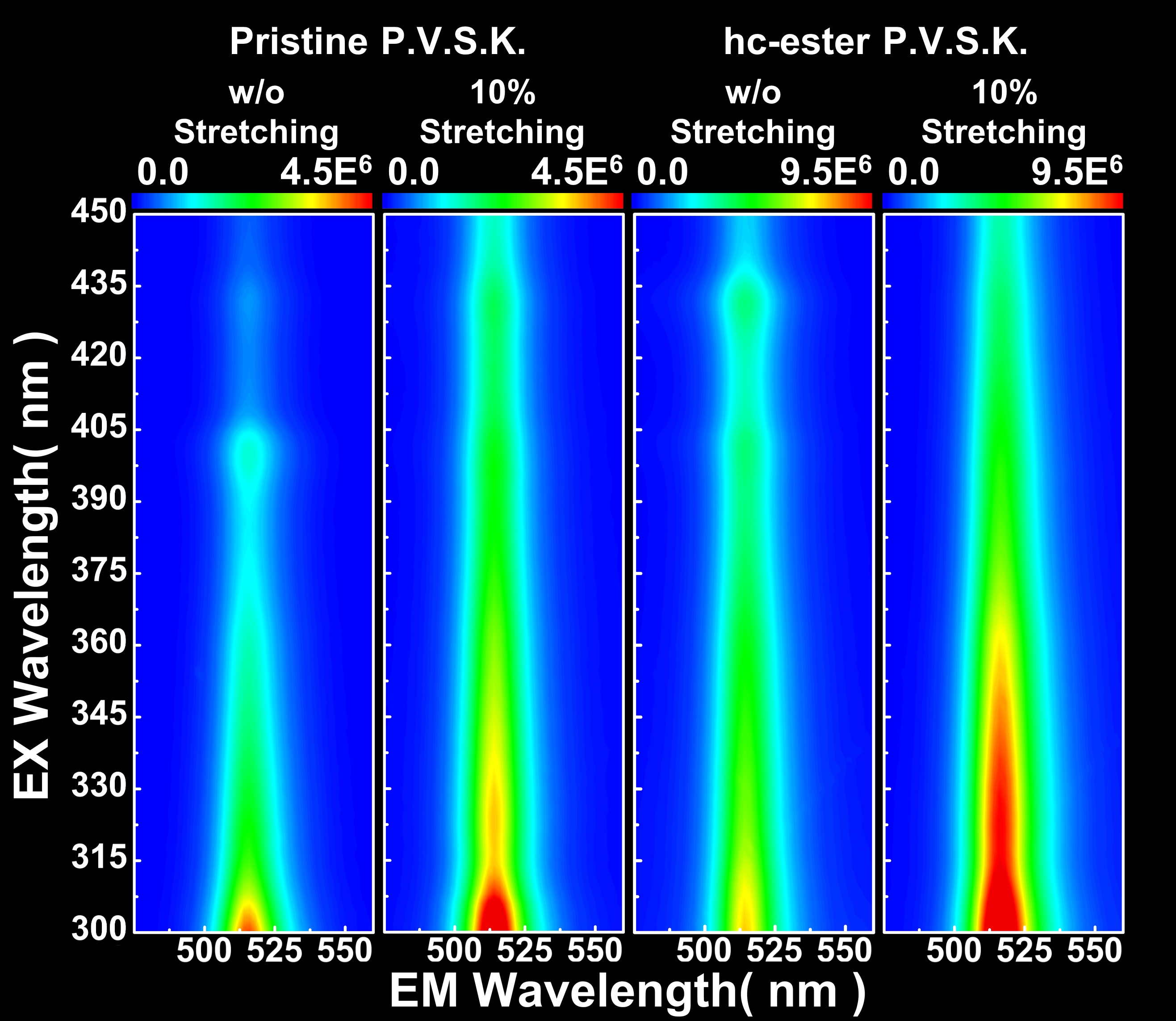


**Fig. S4**. 2D-PL spectra of hc-ester P.V.S.K.@FMRD and pristine P.V.S.K.@FMRD before and after stretching at different excitation wavelengths.


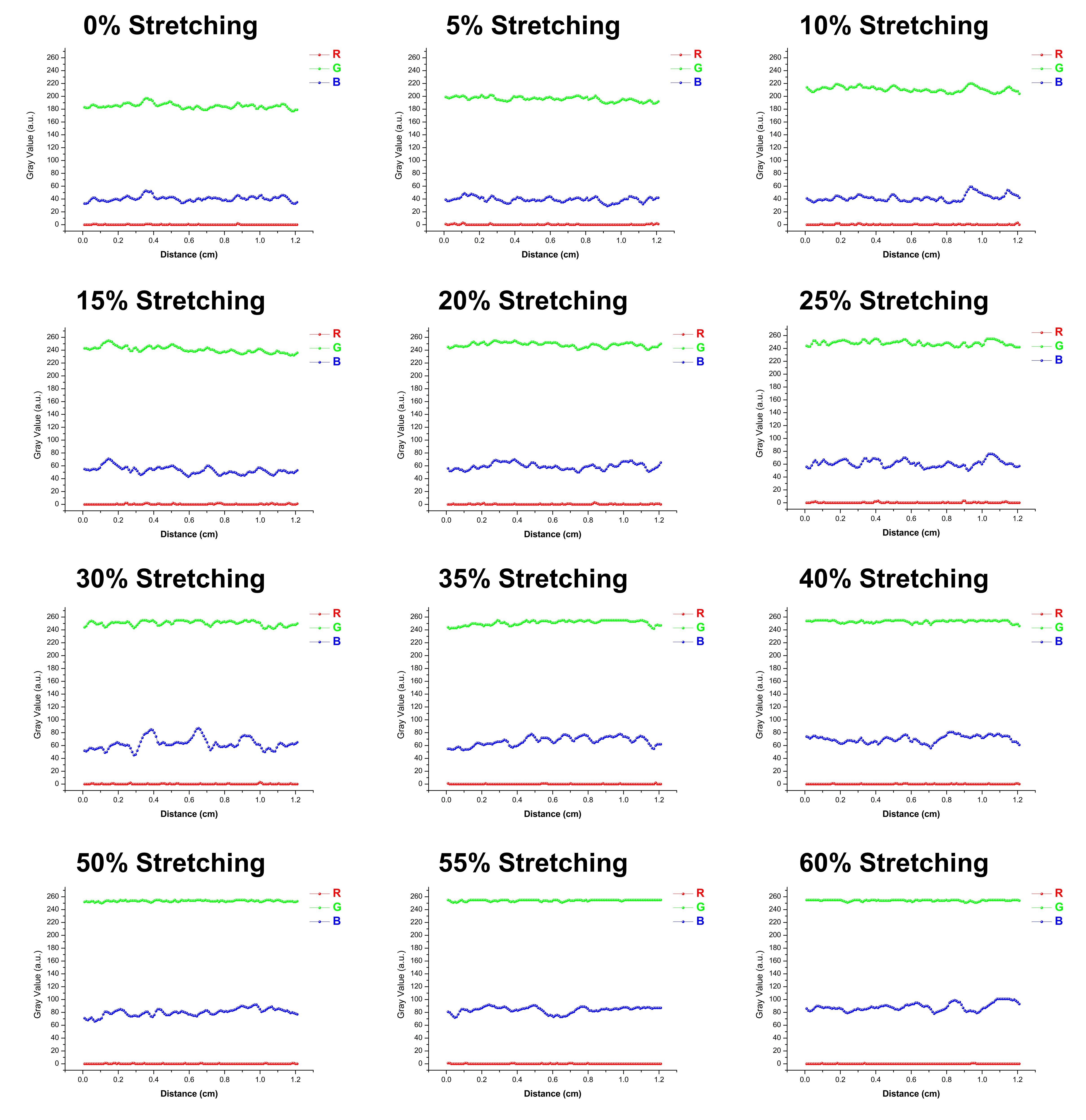


**Fig. S5**. **RGB grayscale profiles of hc-ester P.V.S.K. under different stretching ratios.** Grayscale values of R, G, and B channels were recorded along a 1.2 cm distance under 0% to 60% stretching. G and B intensities increase with strain, while R remains constant as a baseline.


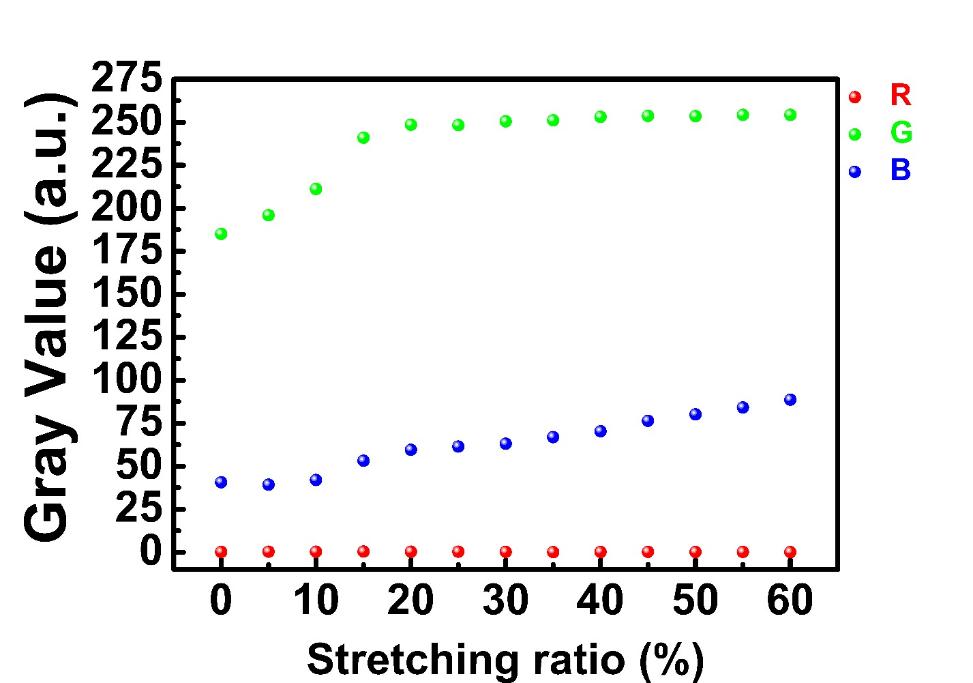


**Fig. S6**. Mean RGB grayscale values of hc-ester P.V.S.K. under stretching.


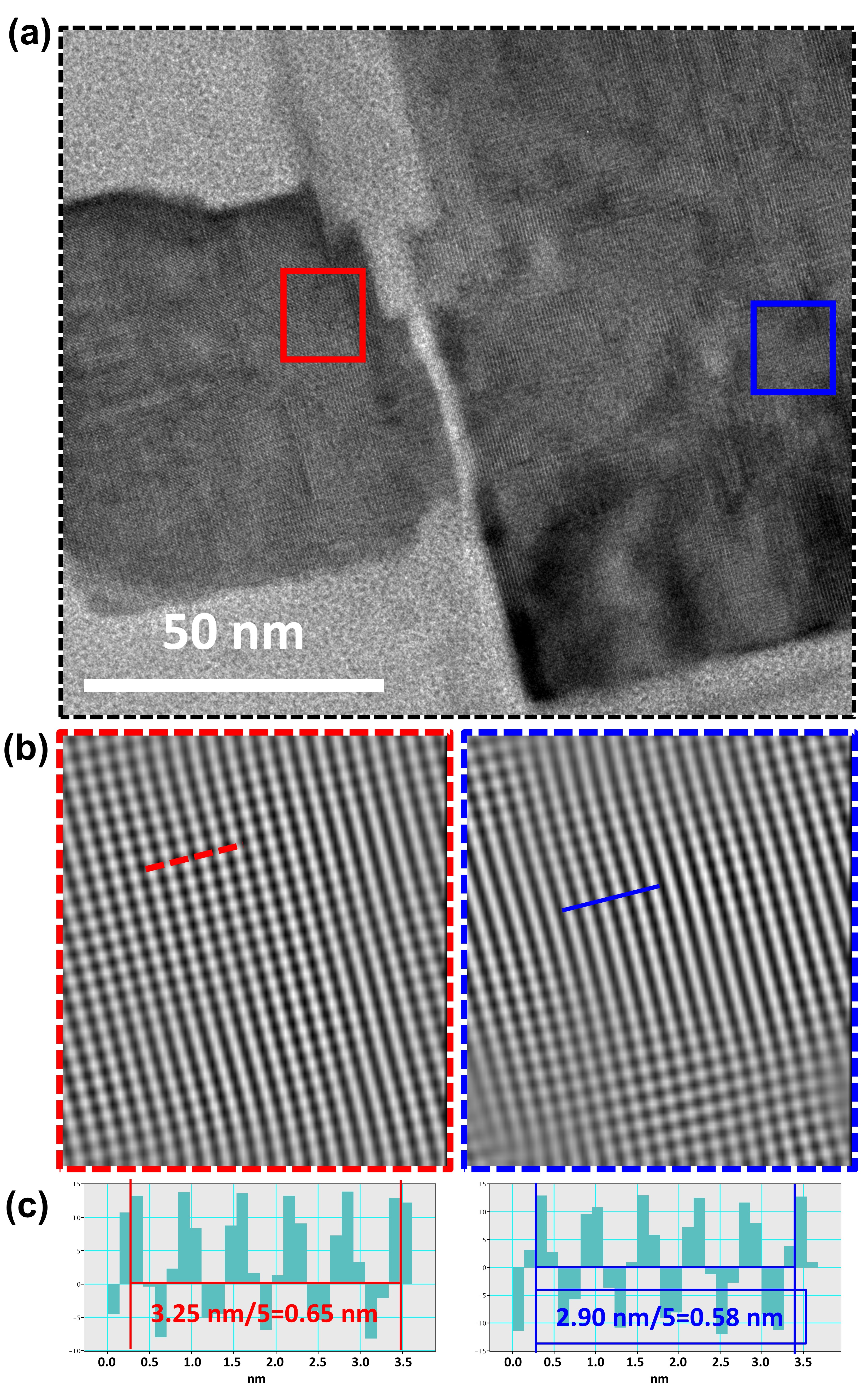


**Fig. S7**. a) TEM image of hc-ester P.V.S.K. showing distinct crystalline domains separated by the chain of the ester polymer. b) Inverse fast Fourier transform images of the red and blue boxed areas in (a), corresponding to low-dimensional (left, red) and high-dimensional (right, blue) P.V.S.K. crystalline domains, respectively. c) d-spacing analysis of the two regions, showing interplanar distances of 0.65 nm (low-dimensional phase) and 0.58 nm (high-dimensional phase).





**Fig. S8**. PL lifetime of hc-ester P.V.S.K. with before and after stretching.


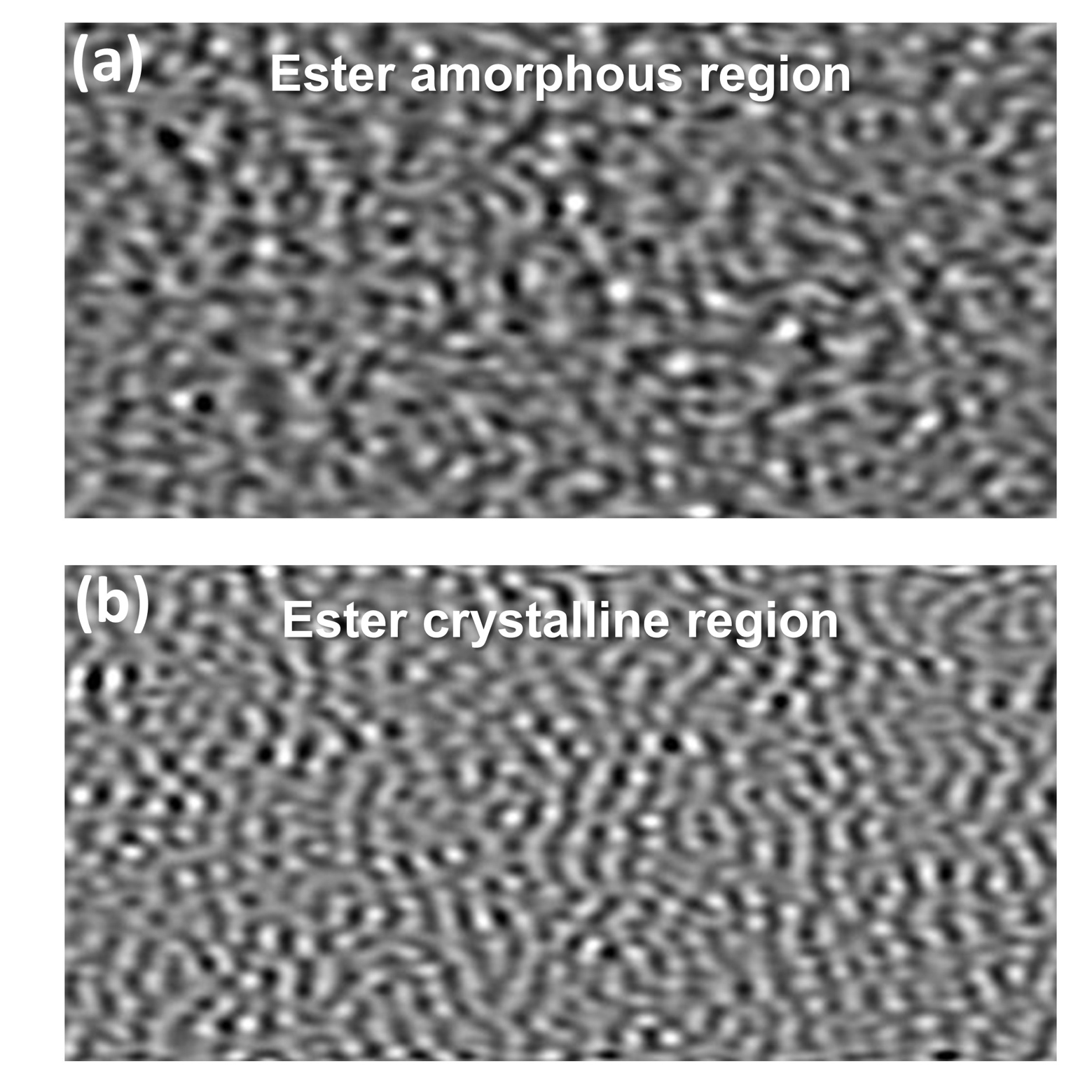


**Fig. S9**. Inverse fast Fourier transform images of the polymer region of a) lc-ester P.V.S.K. and b) hc-ester P.V.S.K.


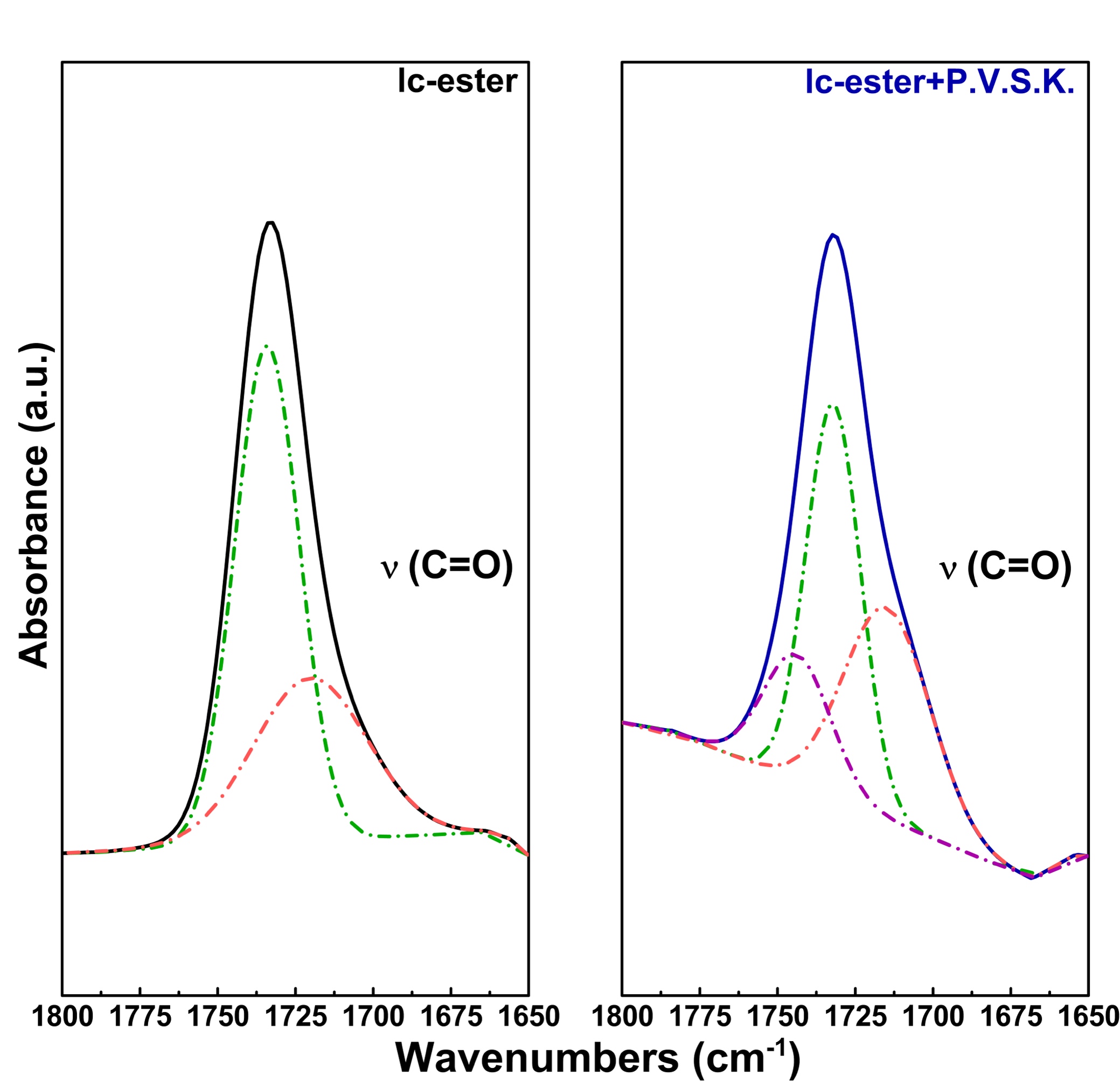


**Fig. S10**. FTIR spectra-ν C=O stretching of lc-ester and lc-ester+P.V.S.K.


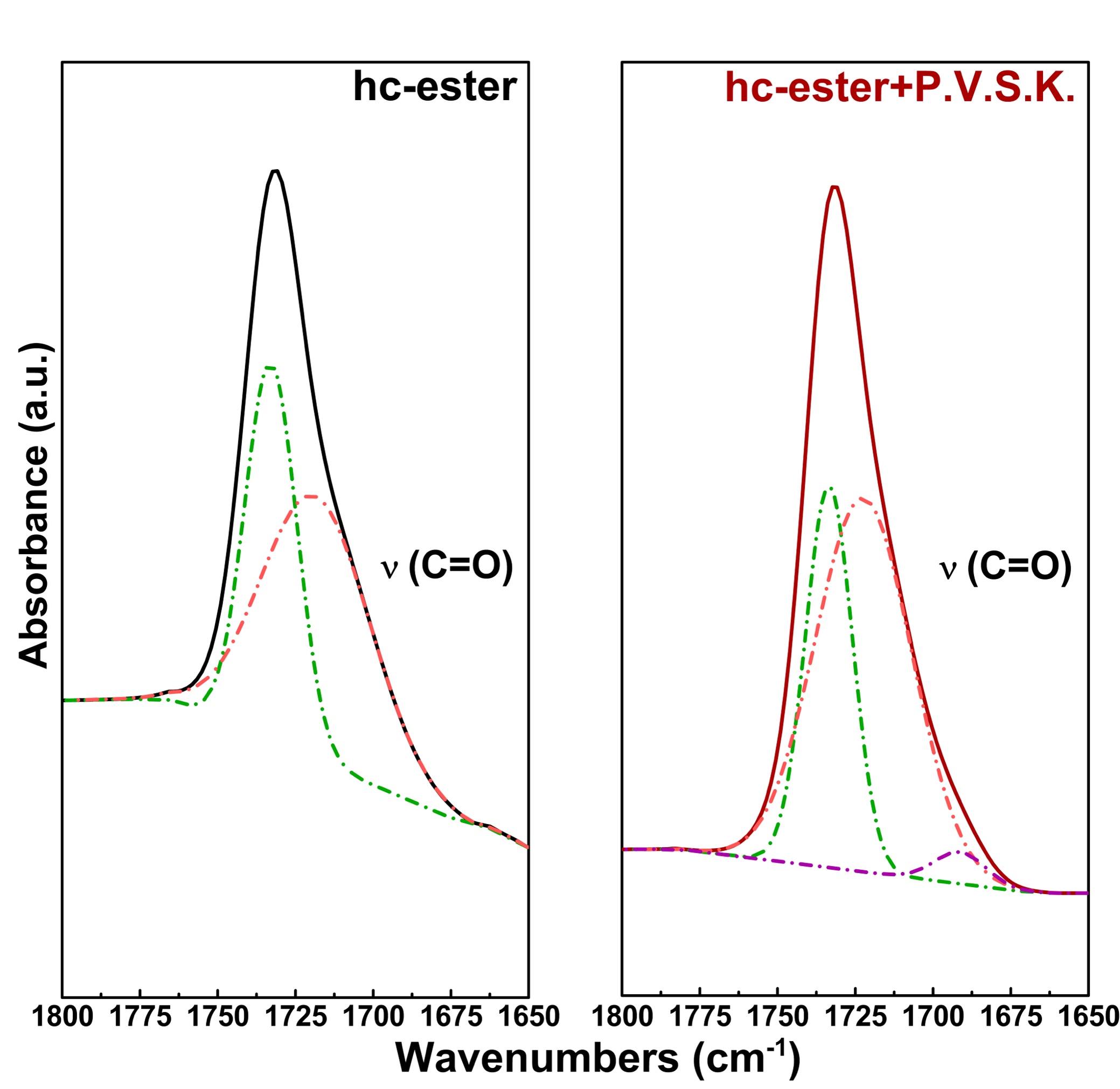


**Fig. S11.** FTIR spectra-ν C=O stretching of hc-ester and hc-ester+P.V.S.K.


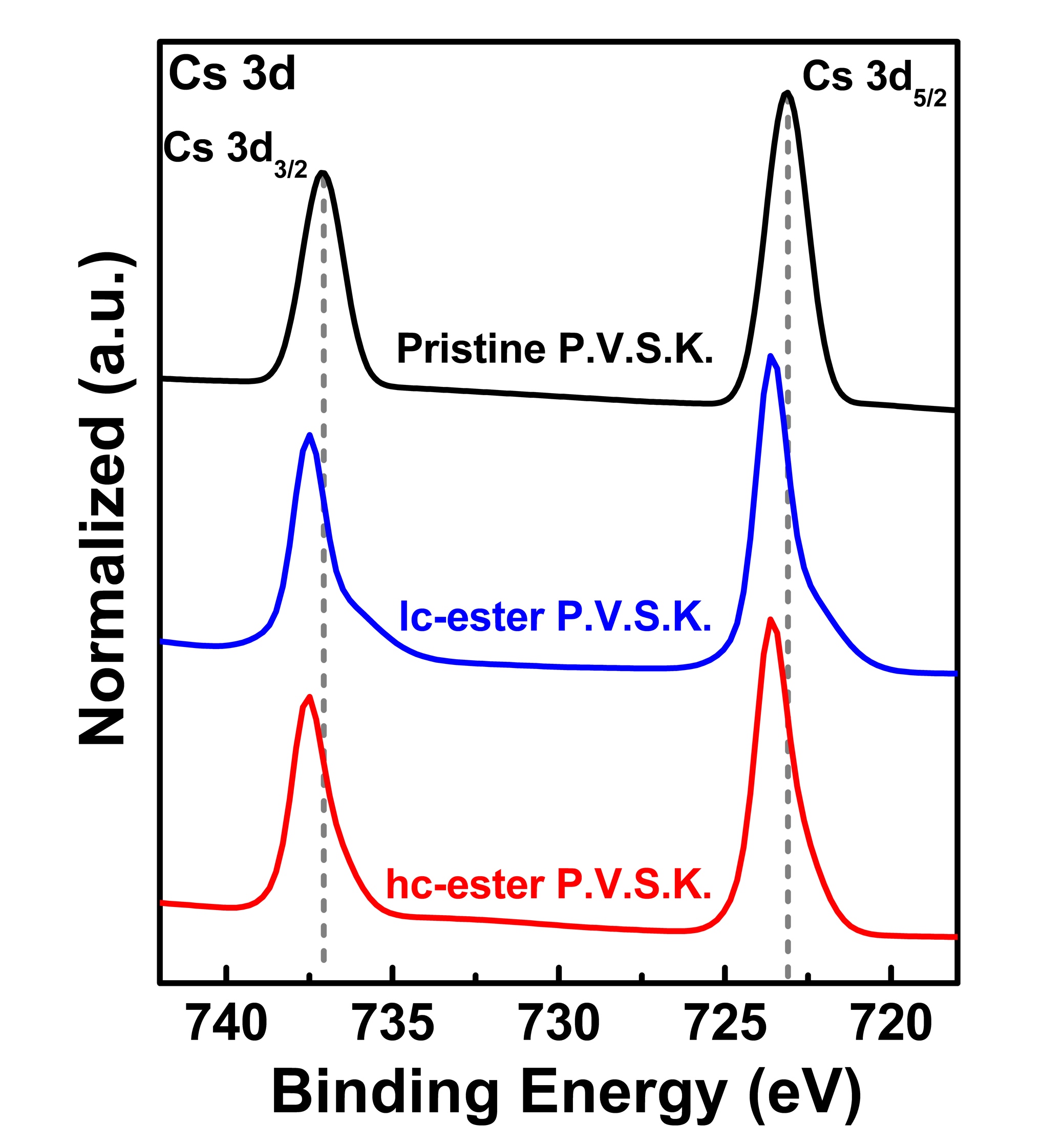


**Fig. S12**. XPS spectra of Cs 3d orbitals.


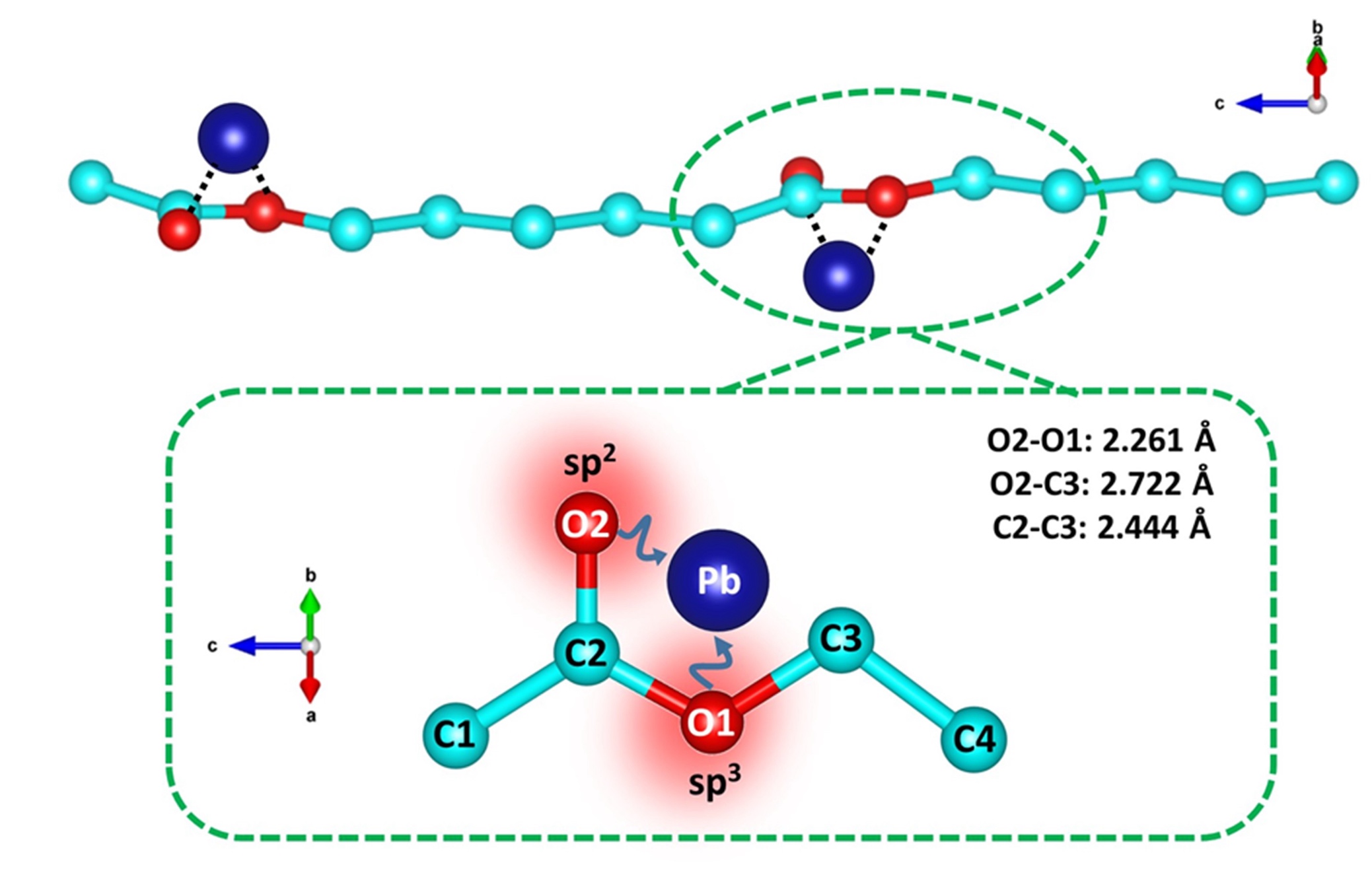


**Fig. S13.** The structure and schematic diagram of the ion-dipole interaction between the ester group and Pb^2+^.


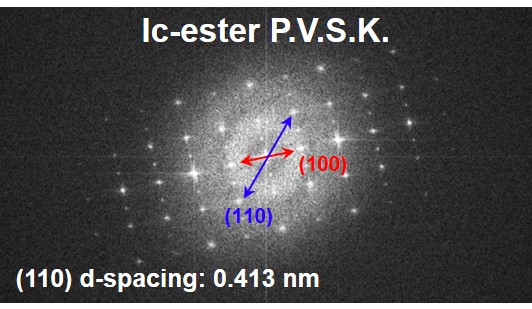


**Fig. S14**. FE-TEM diffraction pattern of lc-ester P.V.S.K.


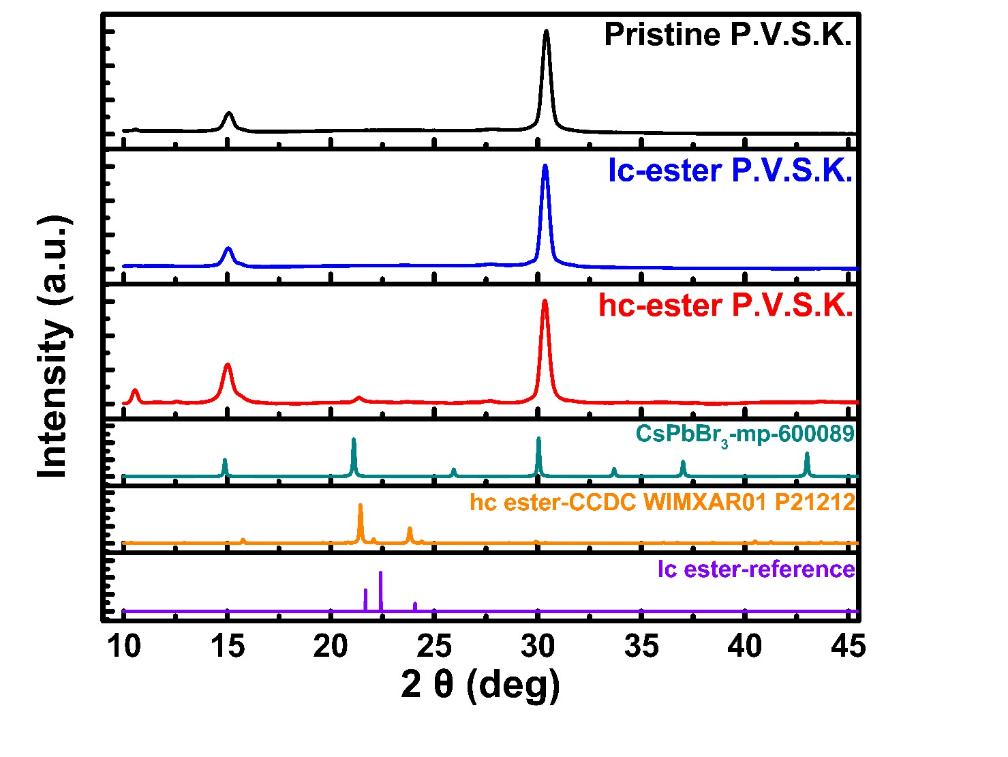


**Fig. S15**. XRD patterns of Pristine P.V.S.K., lc-ester P.V.S.K., and hc-ester P.V.S.K. (References of CsPbBr3, hc ester, le ster ^[S1]^)

+


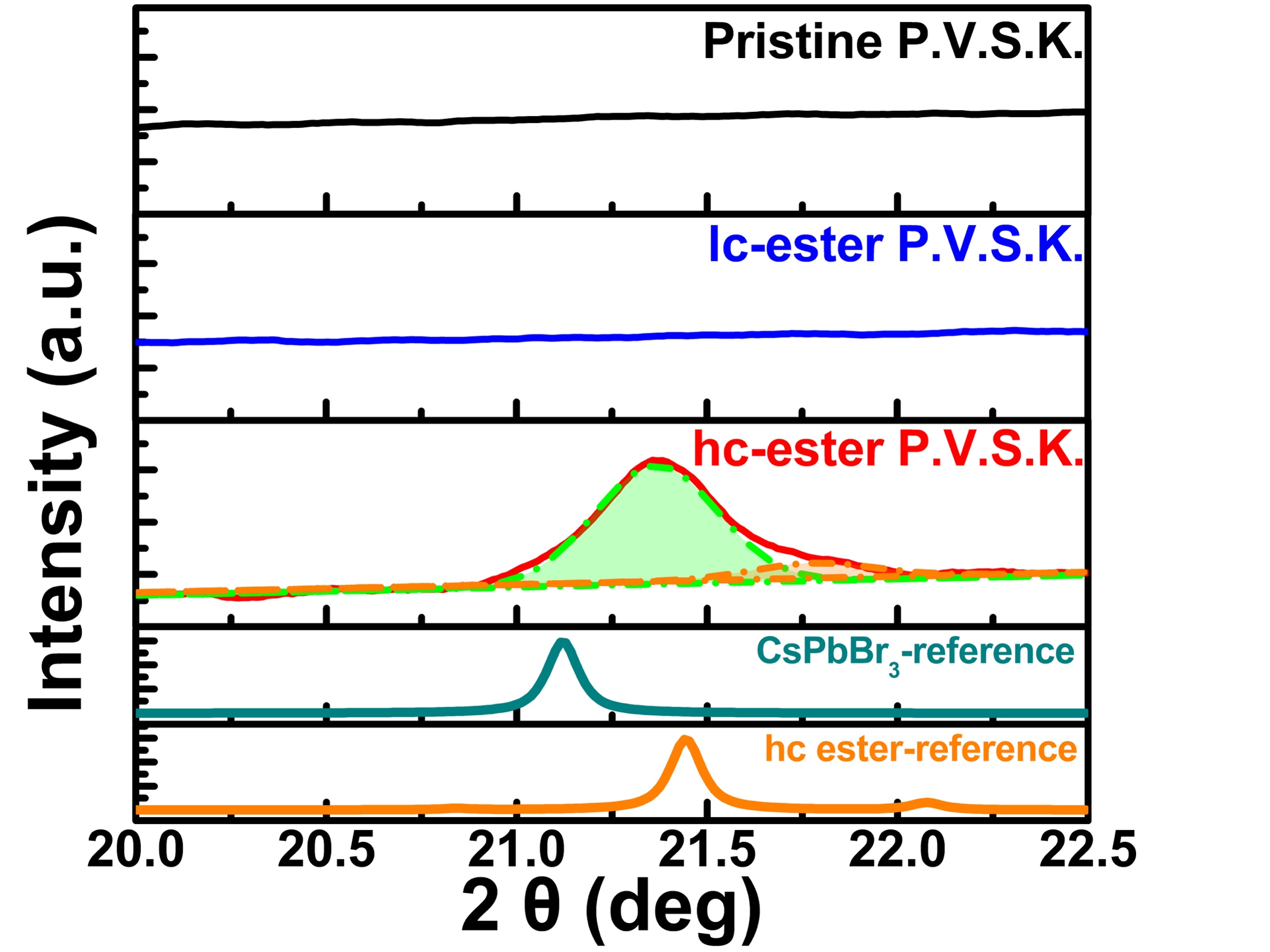


**Fig. S16**. XRD patterns of ester polymer crystallization region of Pristine P.V.S.K., lc-ester P.V.S.K., and hc-ester P.V.S.K.


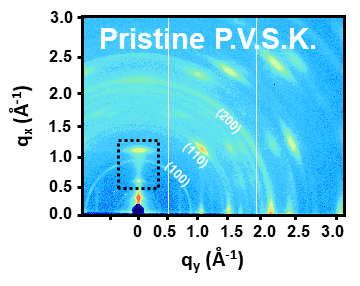


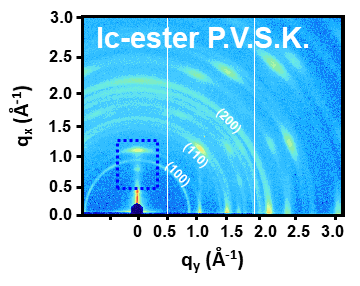


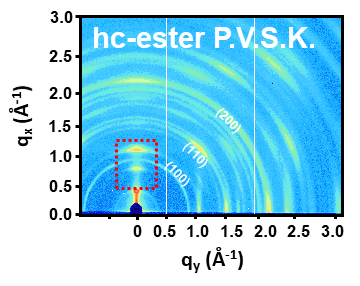


**Fig. S17**. 2D pattern of GIWAXS for Pristine P.V.S.K., lc-ester P.V.S.K., and hc-ester P.V.S.K.


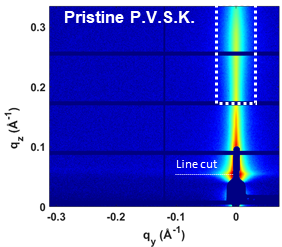


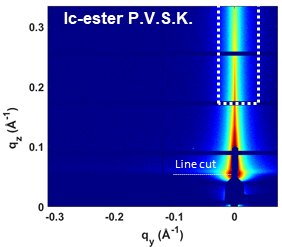


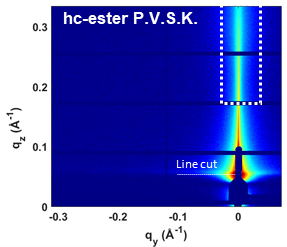


**Fig. S18**. 2D pattern of GISAXS for Pristine P.V.S.K., lc-ester P.V.S.K., and hc-ester P.V.S.K.


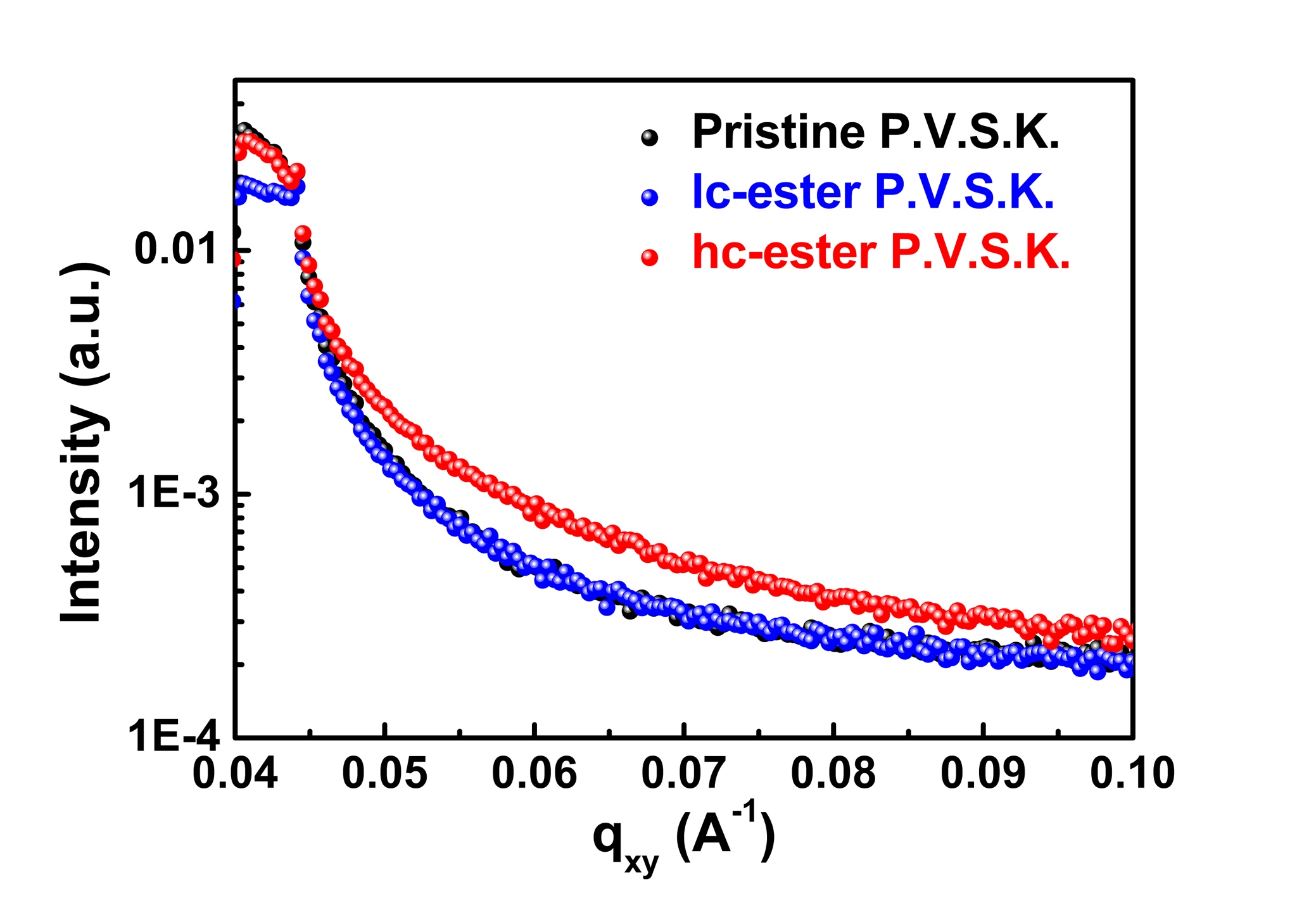


**Fig. S19**. 1D plot of GISAXS for Pristine P.V.S.K., lc-ester P.V.S.K., and hc-ester P.V.S.K.


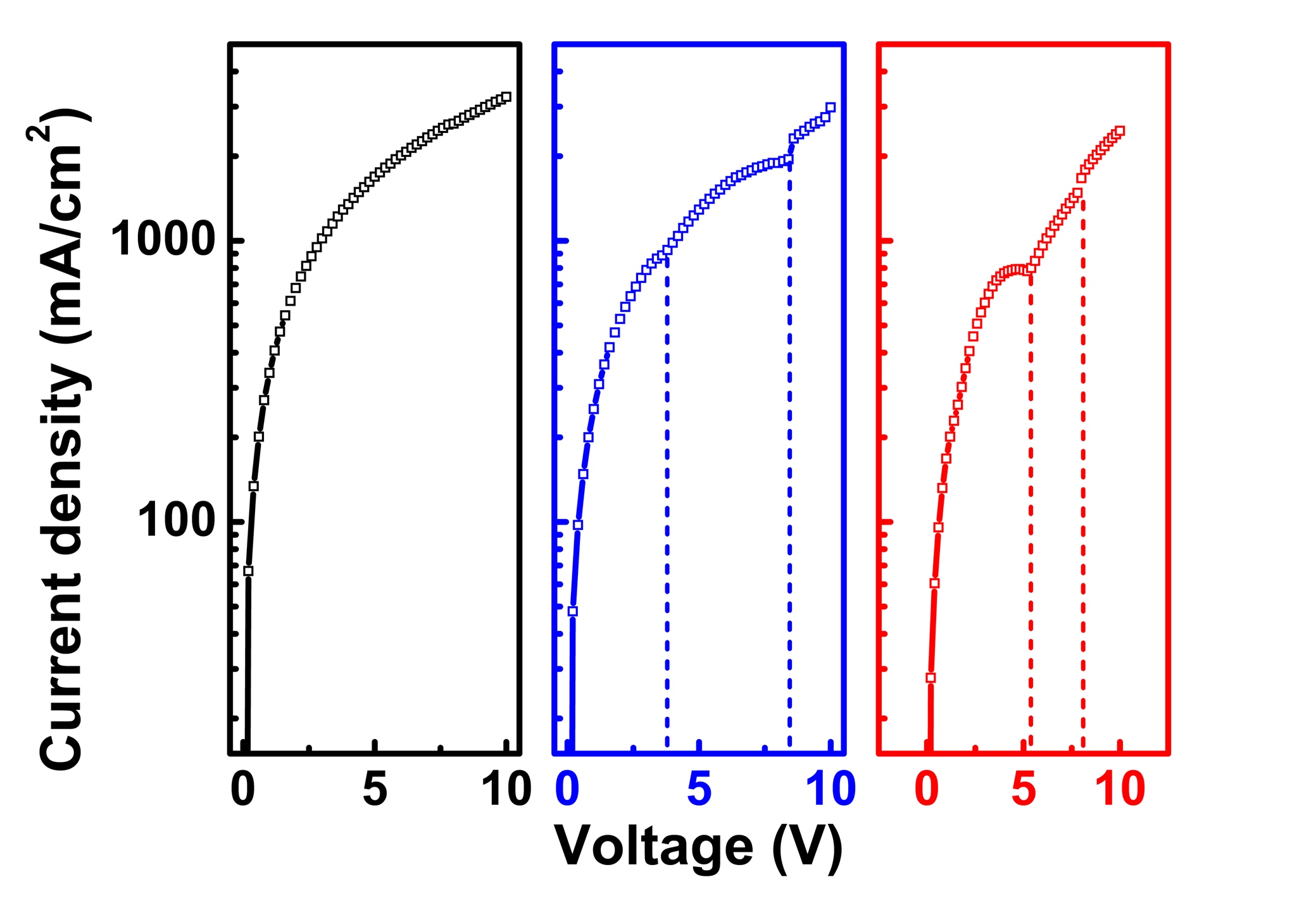


**Fig. S20**. J-V curve of Pristine P.V.S.K.(black), lc-ester P.V.S.K.(blue), and hc-ester P.V.S.K.(red).


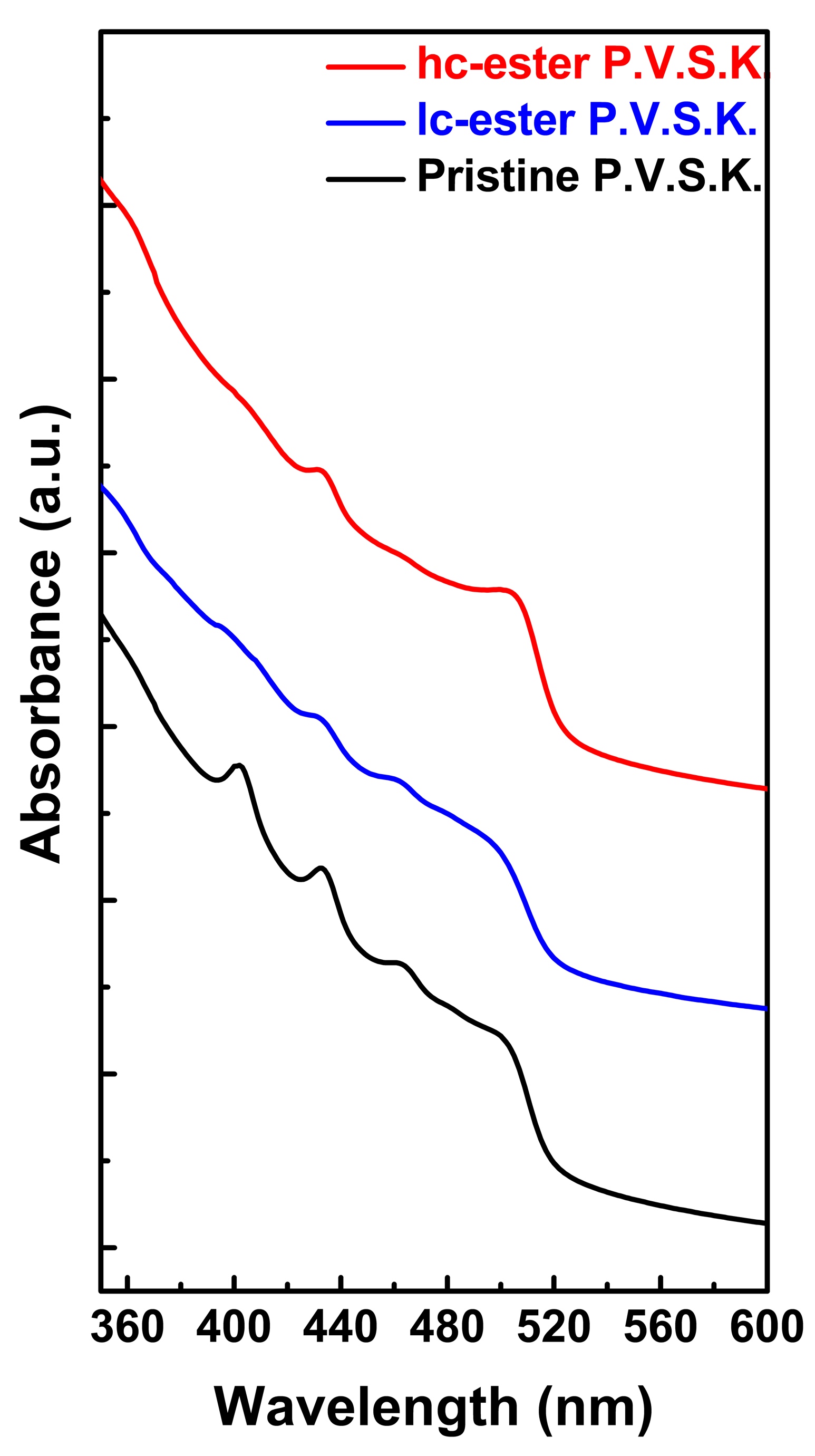


**Fig. S21**. UV-Vis spectrum of Pristine P.V.S.K., lc-ester P.V.S.K., and hc-ester P.V.S.K.


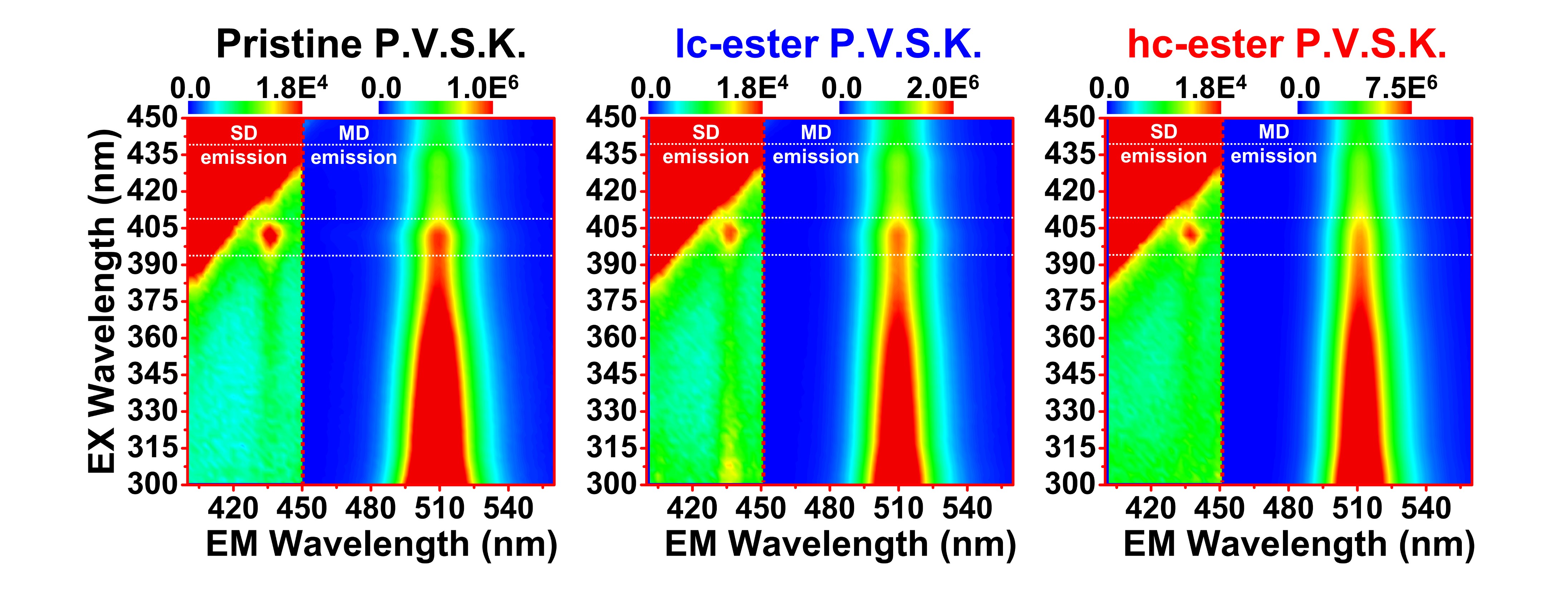


**Fig. S22**. 2D-PL images of SD and MD phase crystals at different excitation wavelengths of Pristine P.V.S.K., lc-ester P.V.S.K., and hc-ester P.V.S.K.


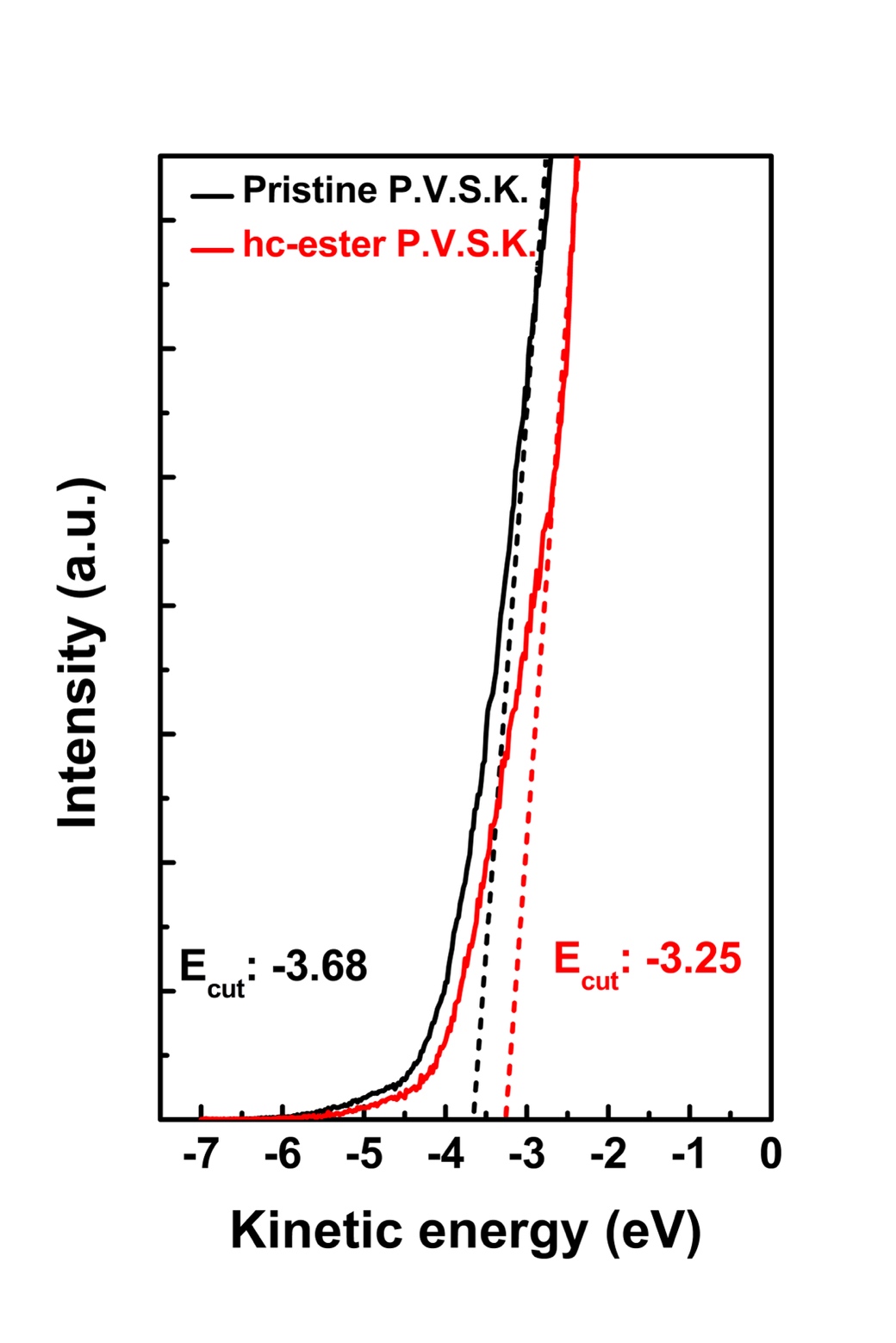

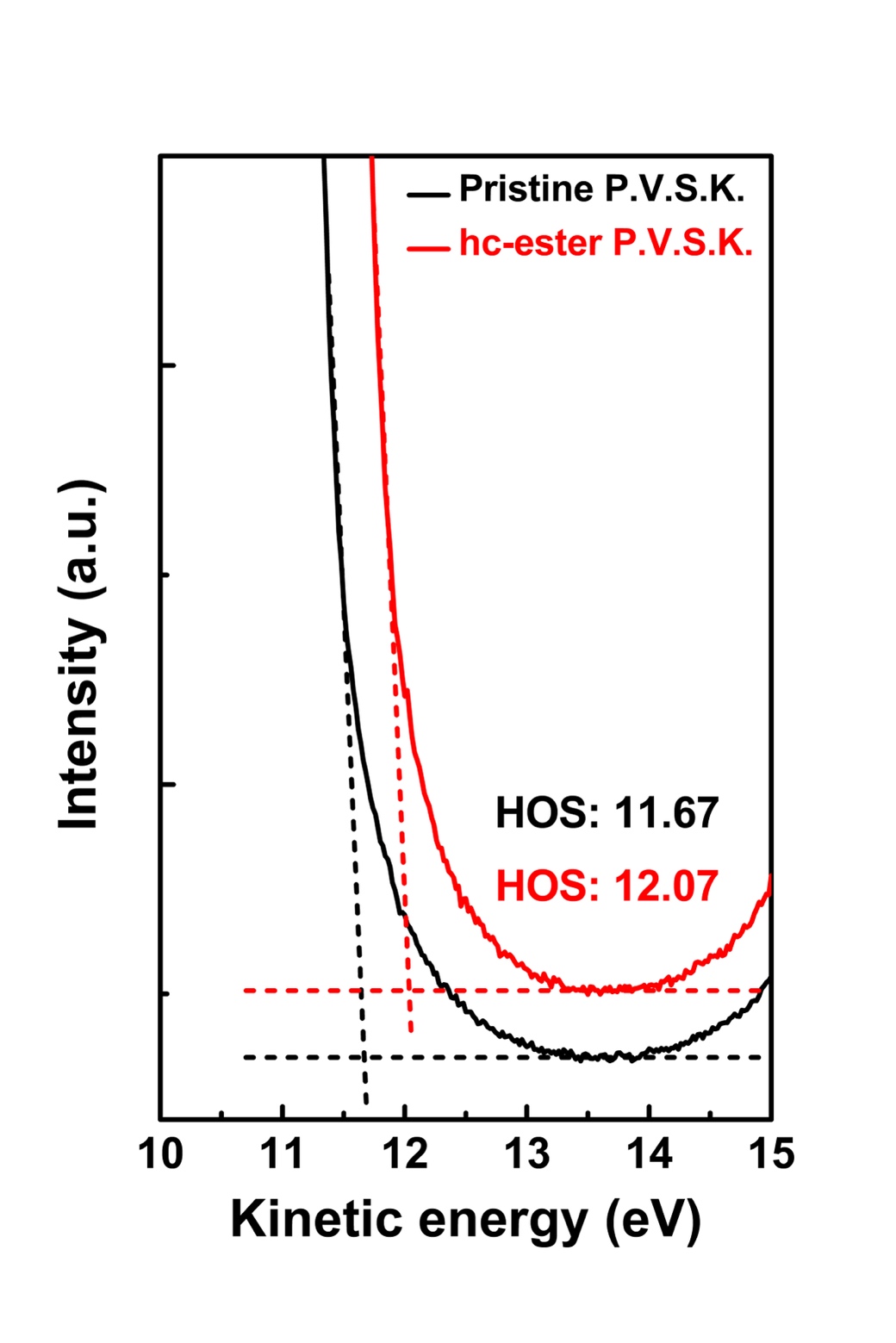


**Fig. S23**. UPS spectrum of Pristine P.V.S.K., lc-ester P.V.S.K., and hc-ester P.V.S.K.





**Fig. S24**. The 1D TD-PL spectra of pristine P.V.S.K. from 300 K to 50 K.





**Fig. S25**. The 1D TD-PL spectra of hc-ester P.V.S.K. from 300 K to 50 K.


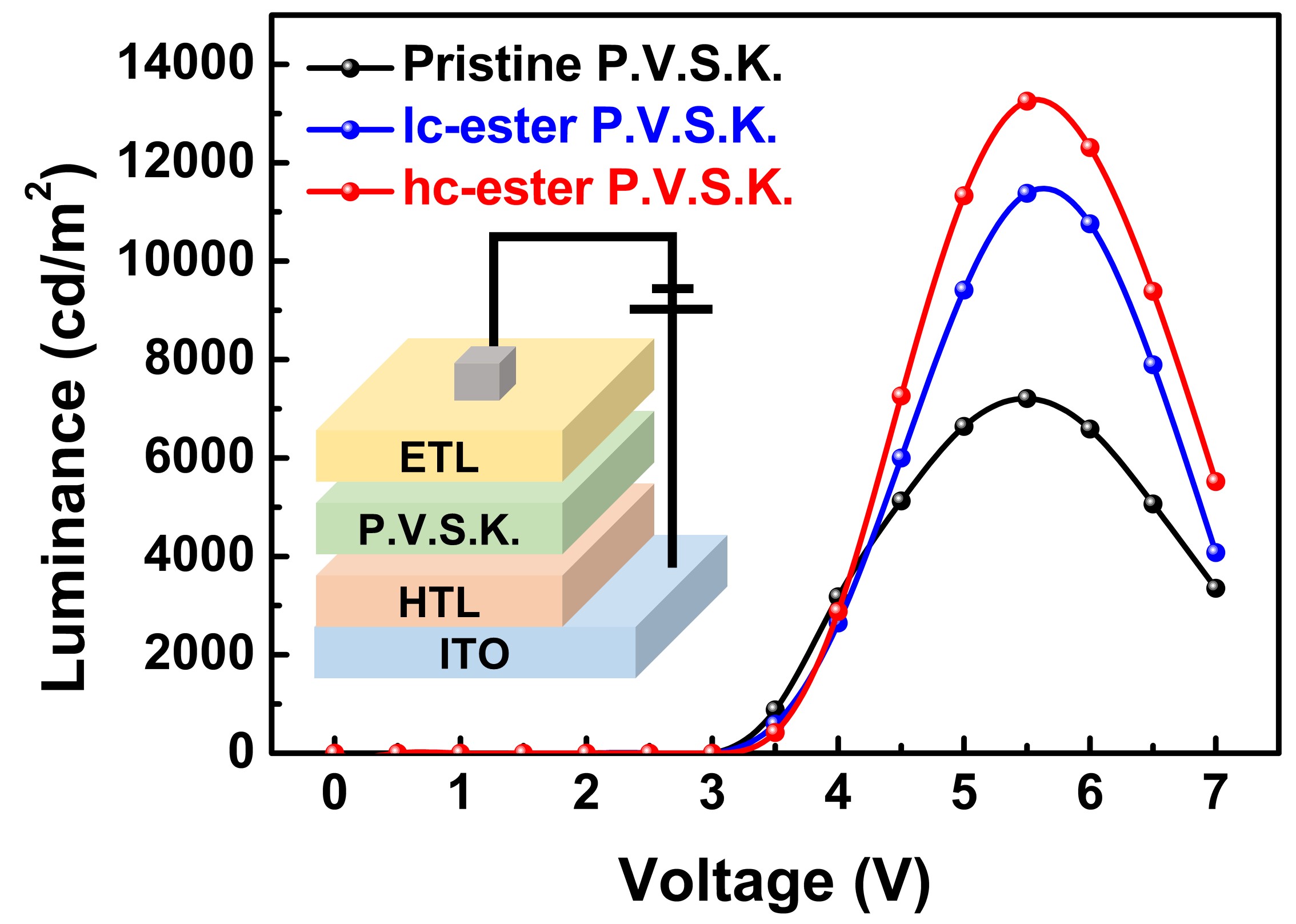


**Fig. S26**. PeLEDs performance - operating voltage versus luminance plot.


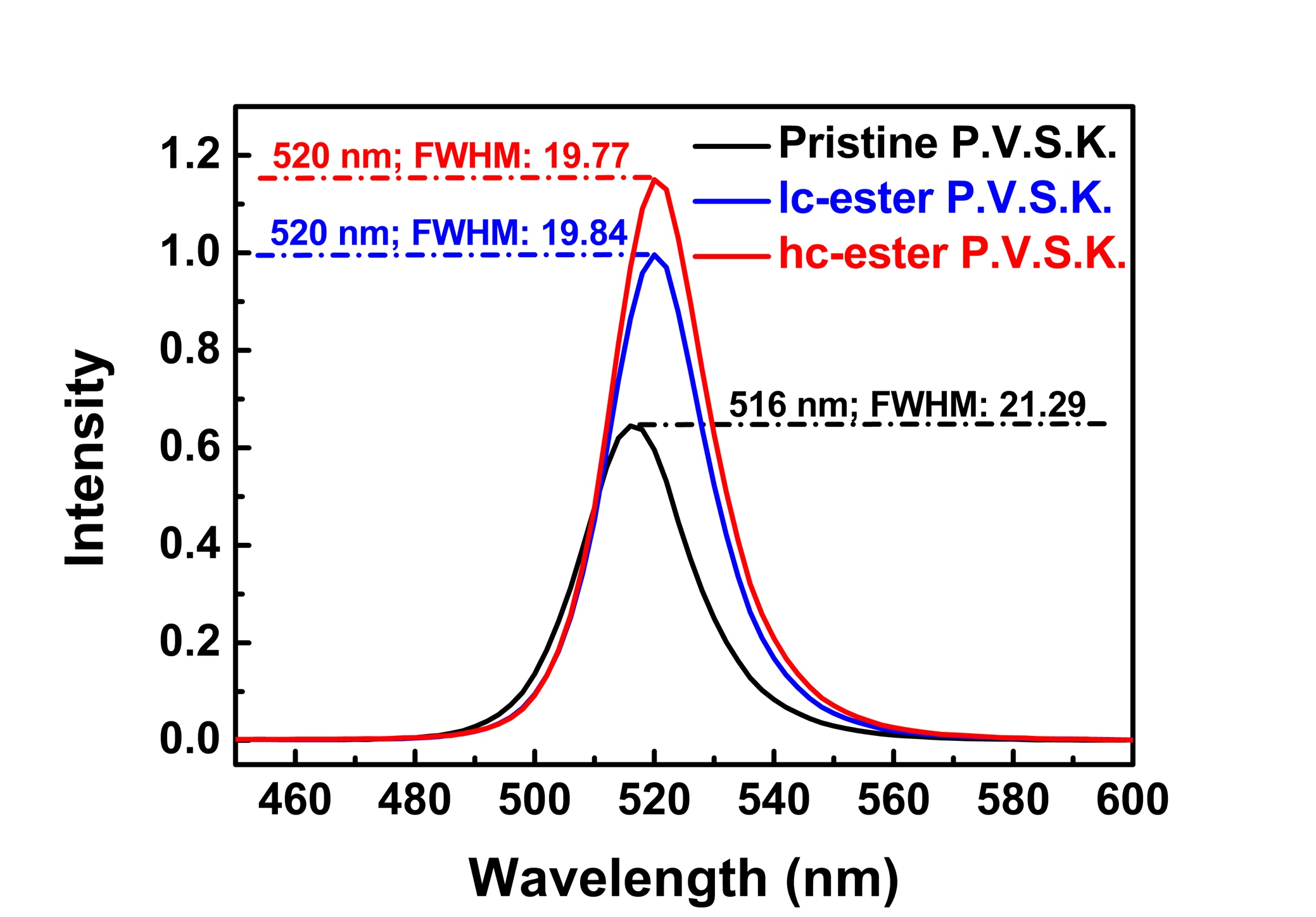


**Fig. S27**. PeLEDs performance - EL spectrum.


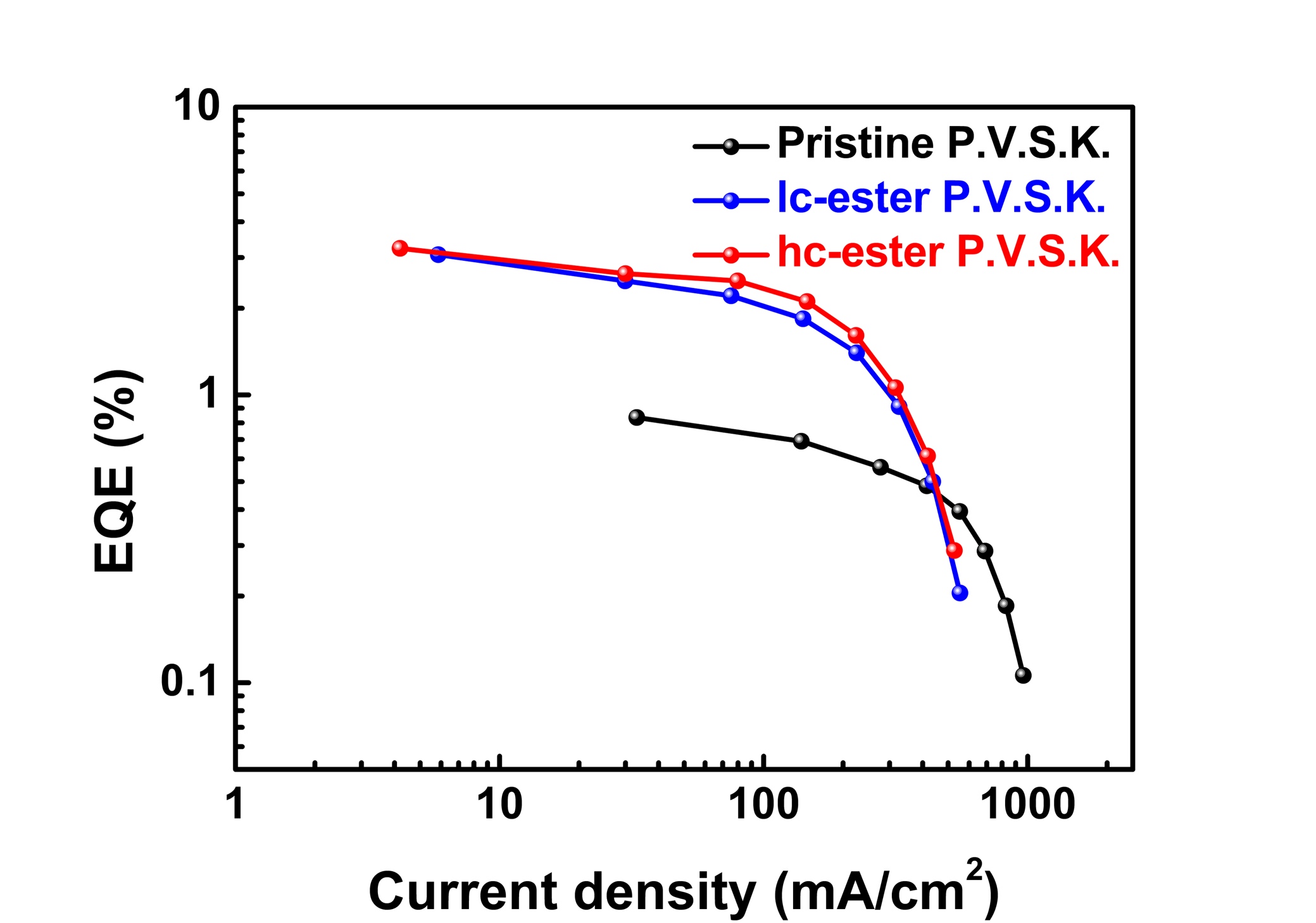


**Fig. S28**. PeLEDs performance – EQE versus current efficiency plot.

**Supplement reference**

[S1] Y.-A., Chen, T.-M., Wu, Crystallization Kinetics of Poly(1,4-butylene adipate) with Stereocomplexed Poly(lactic acid) Serving as a Nucleation Agent. Industrial & Engineering Chemistry Research 2014 53 (43), 16689-16695. https://doi.org/10.1021/ie503303u
